# Active ageing profiles among older adults in Spain: A Multivariate analysis based on SHARE study

**DOI:** 10.1371/journal.pone.0272549

**Published:** 2022-08-04

**Authors:** Fermina Rojo-Perez, Vicente Rodriguez-Rodriguez, Maria-Angeles Molina-Martinez, Gloria Fernandez-Mayoralas, Diego Sanchez-Gonzalez, Jose-Manuel Rojo-Abuin, Alba Ayala, Carmen Rodriguez-Blazquez, Amaia Calderon-Larrañaga, Oscar Ribeiro, Maria-João Forjaz

**Affiliations:** 1 Grupo de Investigacion sobre Envejecimiento (GIE), IEGD, CSIC, Madrid, España; 2 Departamento de Personalidad, Evaluación y Tratamiento Psicológico, Facultad de Psicología, Universidad Nacional de Educación a Distancia (UNED), Madrid, España; 3 Departamento de Geografía, Universidad Nacional de Educación a Distancia (UNED), Madrid, España; 4 Unidad de Analisis Estadistico (UAE), CCHS, CSIC, Madrid, España; 5 Departamento de Estadística, Universidad Carlos III de Madrid (UC3M), Madrid, España; 6 National Centre of Epidemiology and CIBERNED, Carlos III Institute of Health (ISCIII), Madrid, España; 7 Aging Research Center, Department of Neurobiology, Care Sciences and Society (NVS), Karolinska Institutet & Stockholm University, Solna, Sweden; 8 Center for Health Technology and Services Research (CINTESIS), Department of Education and Psychology of the University of Aveiro, Aveiro, Portugal; 9 National Centre of Epidemiology and REDISSEC, Carlos III Institute of Health (ISCIII), Madrid, España; University of Almería, SPAIN

## Abstract

**Background:**

Following the active ageing model based on the Health, Lifelong Learning, Participation and Security pillars, this research has a twofold objective: i) to classify older adults according to active ageing profiles, taking into account the four pillars, and ii) to ascertain the relationship between the profiles and personal and contextual factors, as well as well-being and quality of life in old age.

**Methods:**

A study sample of 5,566 Spanish older adults who participated in wave 6 of the Survey of Health, Ageing and Retirement in Europe (SHARE) was included. Data were analysed in different steps applying several statistical analyses (Principal Component, Cluster, Discriminant, Multiple Correspondence and bivariate analysis with Pearson chi-square and ANOVA).

**Results:**

Five older adult profiles were obtained (I: with moderate activity; II: quasi-dependents; III: with active ageing-limiting conditions; IV: with diverse and balanced activity; V: with excellent active ageing conditions). The first three profiles were characterised by subjects with a high average age, low educational level, who were retired or housewives, and who perceived a moderate level of loneliness, satisfaction with the social network and quality of life, as well as having a larger family network, but living in small households or alone. In contrast, the latter two profiles showed better personal and contextual conditions, well-being and quality of life.

**Discussion and conclusions:**

The multidimensional approach to active ageing followed in this article has revealed the presence of several older adult profiles, which are confined to groups with better or worse active ageing conditions. In this context, if ageing is a process that reflects the previous way of life, intervention priorities will have to consider actions that promote better conditions during the life cycle.

## Introduction and backgrounds

Population ageing is a global phenomenon with important regional differences. Scientific evidence had already pointed to this trend across the board [1]. In Europe, the over-65s represent more than a quarter of the population, having overtaken the number of teenagers and young people aged between 15 and 24 years old [2]. In Spain, the percentage of older adults currently stands at 20%, and is set to keep on rising to 36.8% in 2050 [2].

Different ageing studies take very approaches to the concept, from the pathological approach to an active or successful ageing perspective [3, 4]. The ageing process does not occur randomly, but instead is conditioned by biological, psychological, social and contextual factors that influence ageing trajectories [5]. At older ages, these conditioning factors can trigger health problems, disability and dependence [6], limiting the quality of life of ageing people [7, 8]. The challenge is to fight disease and disability, trying to delay them in order to live independently for as long as possible [9]. The population’s longevity, together with its life expectancy and life trajectory, are key elements for considering that the ageing process is essentially heterogeneous [10]. This vision of old age is reflected in the strategies for promoting positive ageing trajectories [11], which enhance people’s autonomy and independence and their capacities for action as social agents. An example of this is the World Health Organization (WHO) Active Ageing (AA) paradigm [12, 13]. There are also numerous studies that underscore the importance of specific aspects such as learning [14, 15] and the use of information and communication technologies [16] in empowering older adults.

AA is construed as the process of optimising opportunities for Health, Lifelong Learning, Participation and Security in order to improve ageing people’s quality of life [17]. The model, which underpins a wide range of studies [12, 13], identifies determinants such as culture, gender, personal, behavioural and economic factors as well as the physical and social environment and health and social services. General research proves the consistency of a multifactorial model [18]. Different studies base their approach on three key premises: 1) AA is defined as multidimensional, both in its pillars and in the indicators that make up each of them; 2) these dimensions are influenced by a variety of cross-cutting, personal and contextual determinants; and 3) the result of the AA process is an improvement in quality of life [5, 19].

The WHO model-based AA analysis follows three main guidelines. The first emphasises the four pillars, though they are not developed to the same extent, as a result of the different amount of available data used in the quantitative methodology, as well as the inclusion of the Lifelong Learning pillar after the seminal model (2002 versus 2015). Thus, Health is a widely studied pillar [20], which can be used as an enabling factor for AA [21, 22] or as an outcome consistent with an active way of ageing [23–26]. The second pillar, Participation, tends to be very widely represented in any of its meanings, from the most general, engaging in activities [27–30], to other more specific, but highly relevant, activities such as volunteer work [31–36]. Many of these studies refer to participation to highlight its effects on health, well-being and quality of life. Participation is linked to remaining physically active [23], developing social networks [37, 38] or staying on the job market [39] and contributing to productive activities [40]. The other pillars, Lifelong Learning [14, 15] or Security in its different facets [41] do not attract as much progress in the scientific literature, perhaps, again, because the available data do not favour it.

The second guideline is intertwined with the first because certain dimensions are often valued as interactive factors in determining whether a person is actively ageing [42]. Thus, along with the most frequently used purely demographic and individual determinants, such as age and gender [18, 21, 43–45], great importance is attached to others, such as education [46], personal traits of a psychological nature [47], motivation [19, 29] or social and cultural values [46]. Recently, environmental factors for AA, such as collaborative housing [48] or nursing homes as places for care [27], or other factors in the social and political context that explain social inequality, have gained importance [43, 49]. Finally, outcome variables to which AA has been related are also acquiring relevance, such as happiness, life satisfaction, well-being and quality of life [5, 19, 24, 50], to explain regional disparity in Europe according to their social, cultural and institutional differences [23, 45, 51].

In this context, the AA model has become part of a stream of research that explores its capacity to generate types of people and geographical spaces, measuring the degree of activity, by means of quantitative variables [28, 50, 52]. In some cases, specific instruments have been used, such as the AA index as a measure of inequality in general or between countries [45, 53–58]. Other studies have attempted to operationalise the AA model empirically, without reaching a consensus on how to formulate it [59]. At the same time, there are disparities in the types and number of variables and measures and instruments considered in different quantitative models [18, 60, 61] and qualitative approaches [29, 41]. In Europe, the SHARE dataset is becoming a reference framework for the comparative study between countries, especially in its longitudinal perspective and with regard to the study of some of the AA pillars and determinants [62, 63]. In short, and taking into account the AA model’s possible limitations, the importance of its multidimensionality marks a line of research that aims to take a more positive approach [21, 64], identifying groups of older adults according to how they age. In this context, this study has aimed to i) establish profiles of older adults in Spain according to AA pillars, and ii) examine the relationship between these profiles and personal and contextual factors, as well as well-being and quality of life.

## Material and methods

### Data source, study design and sample

This paper has used the Survey of Health, Ageing and Retirement in Europe (SHARE) dataset [63, 65, 66], a representative study of the European countries in which it is carried out. SHARE project became the first European Research Infrastructure Consortium (ERIC) for running a large-scale survey. The survey is reviewed and approved by the Ethics Council of the Max Planck Society, and data collection procedures are subject to continuous ethics review. SHARE-ERIC’s activities related to human subjects research are guided by international research ethics principles such as the Respect Code of Practice for Socio-Economic Research (professional and ethical guidelines for the conduct of socio-economic research) and respects the Declaration of Helsinki in terms of anonymity of the participants and obtaining written consents. Ever since it was created in 2004, SHARE has become an infrastructure for researching and understanding population ageing in Europe from a life-course perspective using a multidimensional and longitudinal approach, although not all countries participate in this network. The target population is anyone aged 50 and over who lives in the country in question on a regular basis. Information is collected through Computer-Assisted Personal Interviewing (CAPI), and is harmonised using a questionnaire that is translated into national languages. Data is collected and produced by modules, and an identifier is used to link data (individuals, households) by wave and/or by module. More information on how the data are collected, eligibility population, sampling procedures and documentation for each participant country, types of respondents, ethical standards, sampling and other technical issues can be seen in the edited book by Börsch-Supan and Jürges [67].

This cross-sectional study is based on wave 6 (SHARE W6), release 6.1.0 (http://www.share-project.org/data-documentation/share-data-releases.html) [68], in which 17 European countries and Israel took part. The fieldwork was completed in 2015 [69]. The data are structured in a set of thematic files, which have been fully reviewed for the selection of the variables of interest in this research. Where available, variables, indices or scales generated by the SHARE project were also used [68]; otherwise, the original variables were used. Participants were retained for Spain (n = 5,566).

In line with the AA model and its pillar structure, variables from all four pillars, (Health, Lifelong Learning, Participation and Security) [17, 42] were used. The authors together reviewed the files, selected the variables and their assignment to the four pillars in order to reach a consensus. Thus, physical, sensory and mental health, functioning, support and health services utilization variables were selected for Health. Lifelong Learning consists of information about educational or training courses and skills. Participation is devoted to information on leisure and community activities participation. Finally, the Security pillar includes variables related to household economics. Variables reporting personal characteristics, area of residence and perceptions of well-being and quality of life, as well as life satisfaction, were also used. For the study of quality of life, we used the CASP-12 scale, a revised and adapted version of the CASP-19 instrument on quality of life in older adults. This is a synthetic indicator, based on the theory of human needs [70, 71], which measures the extent to which older adults’ needs are satisfied in four dimensions: control, autonomy, self-fulfilment and pleasure. The instrument facilitates comparability regardless of the context in which the information is collected [72]. Its discriminatory and explanatory capacity [73] shows that it is an effective tool for measuring quality of life in old age beyond the physical and mental capacities of older adults [74].

The variables selected and used, as well as their characteristics, can be seen in S1 Table. Variables with more than 10% of cases with missing value after weighting were eliminated from the analyses, except for the scale of social connectedness [66, 75] that reported 10.6% of missing cases. Information regarding financial gifts and help was discarded from the analysis due to high floor effect that can lead to biased results [76].

### Statistical analysis

All analyses have been run with SPSS v26. Based on the cross-sectional calibrated weights for individuals, that reproduced the size of the national target population [68], relative or normalised weights have been calculated by dividing the weight by the mean of weights and preserving the sample size [77]. When using the normalised weights, the estimates of means and proportions are correct and the test statistics are not affected.

The variables have been recoded so that more positive conditions take higher values. However, other variables operate in the opposite direction (i.e. the higher the value, the worse the condition) (S1 Table), such as perception of loneliness, units of alcohol consumed, limitations (activities of daily living, instrumental activities, mobility), depression, use of the hospital service (number of times and nights), number of illnesses, number of medications taken, number of technical aids used (such as a cane or walking stick, a zimmer frame or walker, etc.), pain intensity scale, number of reported pains and number of frailty symptoms.

The statistical analysis was carried out in five phases:

- Firstly, the factor analysis technique was applied with the Principal Component Analysis (PCA) extraction method [78] to examine the relationship between the variables selected for the conformation of the AA pillars and reduce their dimensionality. Due to the complexity of the study objective and, especially, the high number of variables required, a PCA was carried out for each thematic set of variables according to the AA pillars (Health, Lifelong Learning, Participation and Security). The factor scores of the 18 Principal Components (PC) of the four AA pillars obtained were retained in the data file to be used in the next analytical phase.
- The second phase consisted of applying Cluster Analysis (CLA) to obtain a homogeneous grouping of older adult subjects according to each of the AA pillars, using the K-Means algorithm, where “k” refers to the number of groups specified a priori by the analyst [78]. Due to the high number of PC, and following the analytical method of grouping variables from the previous PCA, a CLA was performed for each AA pillar. In the Health, Lifelong Learning and Security pillars, the initial cluster centroids were chosen randomly by the programme (default option). However, in the Participation pillar the solution chosen by the programme was not satisfactory, as almost all participants were clustered around the mean. Thus, taking into account the factor structure, a solution was proposed in which the initial centroids were provided so that they saturated in the first four PC of the pillar (PC-11 to PC-14), leaving the last one (PC-15) in the mean (see components of this pillar in S2 Table). The classification obtained by CLA was validated by Discriminant Analysis.
- The clusters resulting from CLA for each AA pillar were used in a third analytical phase to obtain the types of the cluster categories by applying Multiple Correspondence Analysis (MCA). This multivariate method is similar to PCA but for categorical variables, and allows us to ascertain the type of variables from a multidimensional perspective [79]. MCA analyses relationships between variables by representing the categories in a multidimensional space [80], so that the distance between categories is used to establish the degree of similarity and plot a perceptual graph [81], in which proximity between categories indicates association, while remoteness is interpreted as independence.
- Using the MCA category types, the fourth step was to assign each subject or participant in the study to the corresponding AA profile, using the mean of the categories that formed each type in the two MCA dimensions. These means served as centroids in performing a subsequent CLA without centroid updates, so that each subject was assigned to the closest type of categories. This resulted in a classification of subjects by AA profiles to be used in the subsequent analytical phase.
- Finally, to address the second objective of this study, i.e. to determine the relationship between AA profiles and socio-demographic factors and quality of life conditions, bivariate statistical analysis was applied (contingency tables with χ2 test) with categorical variables. Furthermore, with the scale variables, an ANOVA (with Bonferroni test for multiple comparisons) was conducted to compare the AA profiles in each independent variable. Statistical significance levels were set at p < .05.

## Results

### Sample characteristics

The sample consisted of 5,566 participants (Table 1),with a mean age of 67.2 years, (Minimum, Min: 51; Maximum, Max: 106; Mean Standard Error, MSE: 15), 53.8% of whom were women. This sample remained in the education system for an average of 8.8 years (Min: 0; Max: 25; MSE: 0.1), such that 39.4% of the people completed their primary education or the first stage of basic education, and 23% secondary basic education (lower or second stage). As regards activity, 38.8% of the subjects were retired, but almost a quarter remained active, and slightly less than 3 out of 10 were engaged in housework. Two thirds of the older adults were married or living with a partner, and the average household size was 2.2 members.

**Table 1:** Characteristics of the sample.

	Frequency (valid cases)		Descriptive statistics			
Variables and categories	N	%	Min	Max	Mean	MSE
Age in 2015	5566		51	106	67.2	0.15
Gender	5566					
Male	2570	46.2				
Female	2996	53.8				
Years of education	5566		0	25	8.8	0.068
Level of education (based on the ISCED 1997)	5566					
Level 0 –Pre-Primary education	722	13.0				
Level 1 –Primary education or First stage of basic education	2194	39.4				
Level 2 –Secondary basic education (lower or second stage)	1281	23,0				
Levels 3–4 –Secondary education (upper & post)	747	13.4				
Levels 5–6– Tertiary education (first & second)	622	11.2				
Marital status	5566					
Married, living with spouse	3714	66.7				
Registered partnership	76	1.4				
Married, not living with spouse	59	1.1				
Never married	427	7.7				
Divorced	302	5.4				
Widowed	988	17.7				
Current job situation	5521					
Retired	2140	38.8				
Employed or self-employed	1338	24.2				
Unemployed	409	7.4				
Permanently sick	226	4.1				
Homemaker	1250	22.6				
Other	158	2.9				
Household size	5566		1	10	2.4	0.016
Household type	5566					
Single household	1797	32.3				
Household in couple & others	3769	67.7				
Number of children	5566		0	12	2.2	0.02
Number of grandchildren	5566		0	20	2.1	0.037
Area of building	5200					
Rural areas	372	7.2				
Large/small towns	3262	62.7				
A big cities/metropolitan areas	1566	30.1				
Type of building	5231					
Farm/family house/double house	2527	48.3				
Building with 3+ flats or high-rise	2670	51,1				
Housing with services for elderly/nursing home	34	0.6				
Housing tenure regime	5521					
Owner	5103	92.4				
Others (cooperative, tenant, subtenant, rent free)	418	7.6				
Loneliness (short version of R-UCLA Loneliness Scale) (high is lonely)	5213		3	9	3.7	0.019
Social network satisfaction (high is more satisfaction)	5252		0	10	8,9	0.017
Life satisfaction (high is more satisfaction)	5566		0	10	7.5	0.025
CASP-12 index for Quality of Life and Wellbeing (high is better QoL)	5087		12	48	36.1	0.091

In shadow, scale variables.

Min: Minimum; Max: Maximum; MSE: Mean Standard Error.

In residential environment terms, almost two thirds of the older adults resided in large or small towns and half of them occupied dwellings in block buildings (3 or more flats), though more than 4 out of 10 reported living in a block building.

As for other living conditions, older adults showed a mean loneliness score of 3.7 (Min: 3; Max: 9; MSE: 3.7) (the higher the index, the higher the loneliness), and a mean quality of life score (CASP-12) of 36.1 (Min: 12; Max: 48; MSE: 36.1) (higher numbers implying better quality of life). The level of satisfaction with life in general and with the social network obtained mean values of 7.5 and 8.9, respectively (measured on a scale from 0: completely dissatisfied to 10: completely satisfied).

### Investigating the relationship among the variables

The PCA performed for each AA pillar provided 18 PC (S2 Table). The Health Pillar-related PCA was formed by 8 PC, explaining 65.7% of the variance. The Lifelong Learning showed 2 PC explaining an 81.2% of the variance. In the Participation, 29% of the participants had no complete information, thus each variable with missing values was replaced with the mean of the variable; 5 PC were retained explaining a 69.9% of the variance. Finally, in the Security Pillar formed by economic variables, an overall 69.3% of the variance was explained by 3 PC.

### Grouping participants based on the active ageing pillars

Applying CLA with the PC for each AA pillar resulted in 17 clusters (Table 2).

**Table 2:** Cluster analysis through k-means method.

	Cluster and final cluster centres				
Principal Components of Health Pillar	H-1: Need of help for functioning	H-2: Moderate health	H-3: Bad health	H-4: Unhealthy habits	H-5: High hospital use
PCH-1: Bad health	**0.456**	**-0.425**	**1.271**	-0.117	-0.053
PCH-2: Bad functioning	**0.385**	-0.247	-0.210	0.148	**0.515**
PCH-3: Good cognitive functioning	-0.187	0.138	0.091	-0.139	**-0.550**
PCH-4: High use of hospital services	-0.288	-0.133	**1.725**	-0.162	**7.687**
PCH-5: Good sensory health	-0.216	0.079	-0.215	**0.464**	0.253
PCH-6: Do not need help / Do not use of technical aids for activities	**-0.938**	**0.582**	0.293	-0.159	**-0.427**
PCH-7: High protein diet	-0.066	0.014	-0.043	0.209	-0.218
PCH-8: High green-dairy/Low alcohol consumption	0.296	0.236	0.095	**-2.537**	0.135
Number of Weighted Cases in each Cluster (valid cases: 5555) N (%)	1788 (32.2)	2865 (51.5)	365 (6.6)	492 (8.9)	45 (0.8)
98,2% of original grouped cases correctly classified.					
	Cluster and final cluster centres				
Principal Components of Learning Pillar	L-6: Low competence	L-7: Competence & training involvement	L-8: High competence	L-9: High training involvement	
PCL-9: Good writing/reading/ICTs Skills	**-0.743**	**0.558**	**0.865**	0.011	
PCL-10: Educational-training involvement	-0.219	**1.692**	-0.205	**5.145**	
Number of Weighted Cases in each Cluster (valid cases: 5184) N (%)	2680 (51.7)	165 (3.2)	2193 (42.3)	147 (2.8)	
99,0% of original grouped cases correctly classified.					
	Cluster and final cluster centres				
Principal Components of Participation Pillar	P-10: Low social connectedness / Moderate volunteering	P-11: Low physical & moderate social-political activities	P-12: Physical activities / Social connectedness	P-13: Cognitive activities	
PCP-11: High frequency of cognitive activities performance	-0.320	-0.250	**-0.420**	**1.750**	
PCP-12: High frequency of physical activities performance	0.090	**-1.390**	**0.830**	0.160	
PCP-13: High frequency of social & political activities involvement	-0.260	**0.590**	-0.010	-0.200	
PCP-14: High frequency of volunteering activities performance	**0.420**	-0.190	-0.210	-0.290	
PCP-15: High social connectedness	**-0.660**	0.180	**0.660**	0.020	
Number of Weighted Cases in each Cluster (valid cases: 5566) N (%)	1938 (34.9)	1164 (20.9)	1570 (28.2)	893 (16.0)	
96,5% of original grouped cases correctly classified.					
	Cluster and final cluster centres				
Principal Components of Security Pillar	S-14: Optimal household economy	S-15: Self-assessed high economic status	S-16: Self-assessed low economic status	S-17: High value of non-liquid assets	
PCS-16: High self-perception of the household economic status	-0.855	**0.979**	**-0.609**	-1.560	
PCS-17: High value of household non-financial/non-liquid assets & expenditure	-0.346	0.054	-0.054	**11.790**	
PCS-18: High household economic health	**4.565**	0.005	-0.247	-0.899	
Number of Weighted Cases in each Cluster (valid cases: 5521) N (%)	172 (3.1)	2150 (38.9)	3190 (57.8)	10 (0.2)	
99,6% of original grouped cases correctly classified.					

Notes: PC: Principal Component. Clusters are numbered correlatively through all clusters obtained in the analysis: H: Cluster of Health Pillar. L: Cluster of Learning Pillar. P: Cluster of Participation Pillar. S: Cluster of Security Pillar.

- The CLA performed over the Health Pillar’s (H) main components resulted in 5 clusters: H-1: Need of help for functioning (grouped 32.2% of the subjects); H-2: Moderate health (51.5%); H-3: Bad health (6.6%); H-4: Unhealthy habits (8.9%); H-5: High hospital use (0.8%). Consumption of a protein diet and alcohol is a main component that did not stand out in any of the homogeneous groups, as in all of them it is around the mean. Based on Discriminant Analysis we observed that 98.2% of originally grouped participants were correctly classified.
- The CLA performed over the Lifelong Learning Pillar’s (L) main components grouped subjects in 4 clusters: L-6: Low competence (classified 51.7% of cases); L-7: Competence and training involvement (3.2%); L-8: High competence (42.3%) as the opposite group to L-6; L-9: High training involvement (2.8%). 99% of originally grouped participants were correctly classified.
- The CLA performed over the Participation Pillar resulted in 4 clusters (P), named as follows: P-10: Low social connectedness / moderate volunteering (34.9% of cases); P-11: Low physical & moderate social-political activities (20.9%); P-12: Physical activities / social connectedness (28.2%); P-13: Cognitive activities (16.0%). The 96.5% of originally grouped participants were correctly classified.
- The CLA performed over the Security Pillar’s main components captured 4 clusters: S-14: Optimal household economy (classified 3.1% of cases); S-15: Self-assessed high economic status (38.9%); S-16: Self-assessed low economic status (57.8%) contrasts with the previous group; S-17: High value of non-liquid assets (0.2%). 99.6% of originally grouped participants were correctly classified.

### Active ageing profiles

**Fig 1:**
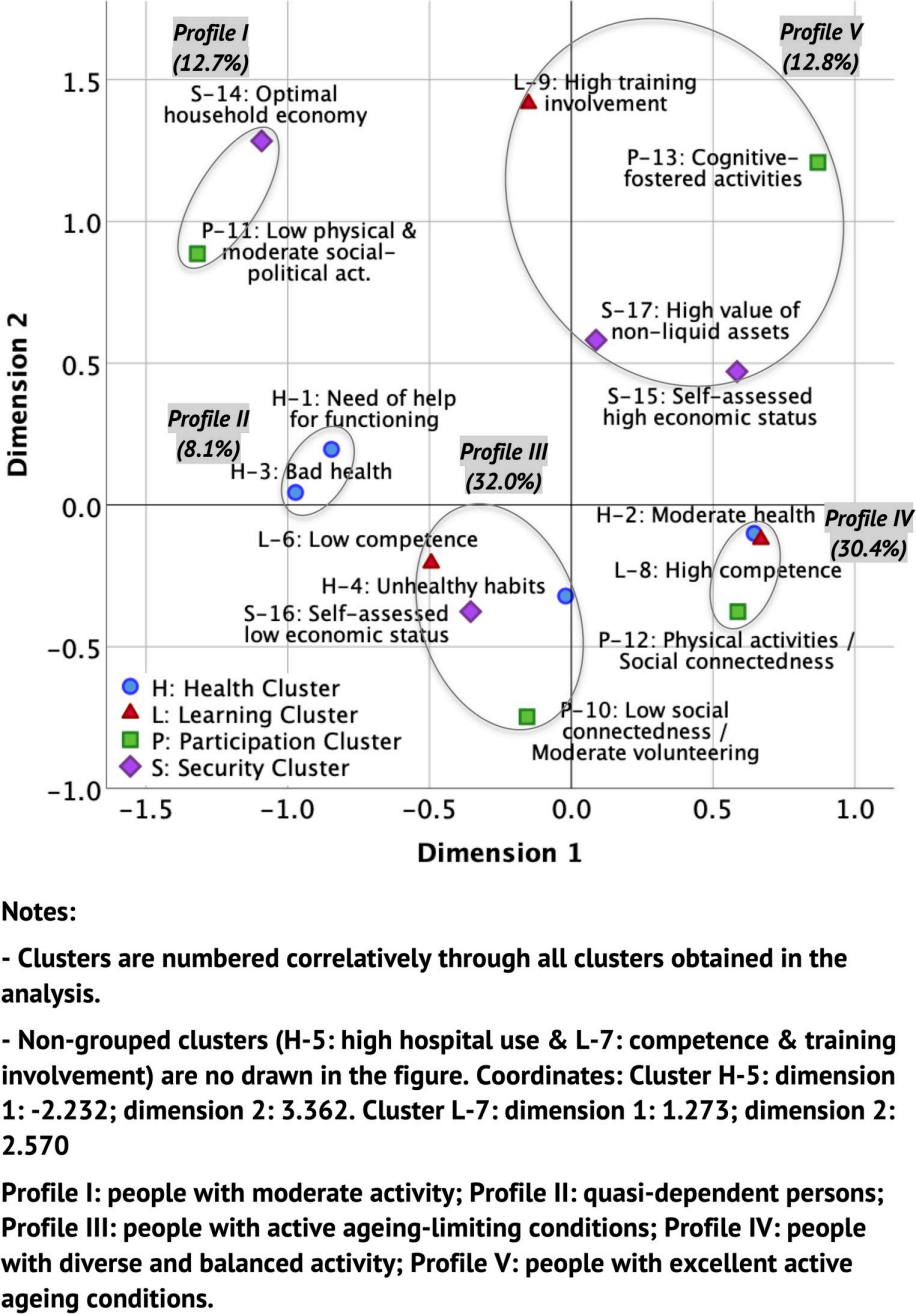
Plot of category clusters.

The MCA gave a perceptual map with the solution obtained in the clustering of profiles according to their AA pillar-related living condition (Fig 1), and shows the distribution of the cluster categories on the plane formed by the coordinate axes. The figure showed two dimensions with eigenvalues, or part of the variance explained in each dimension, higher than 1 (dimension 1: 1.721; dimension 2: 1.245) and together they accounted for 37.1% of the model variability. The relationship of the categories on the plane shows several different subject profiles which, by convention, have been listed starting with the lowest dimension 1 or x-axis value, resulting in a counter-clockwise grouping. The first three profiles are to be found on the lowest scores of dimension 1, as opposed to the rest of the profiles, to be found on the positive values.

**Profile I** (people with moderate activity) included subjects with a low participation in physical activities, together with a moderate frequency of social and political tasks, and at the same time an optimal economic assessment of their household according to the level of income.

**Profile II** (quasi-dependent persons) was characterised by poor health and functioning conditions and frailty and, consequently, by the use of health services (primary and hospital care), and the need for help from others in activities of daily living, as well as the use of technical aids. This profile is only associated with the Health pillar.

**Profile III** (people with active ageing-limiting conditions) grouped subjects with low self-assessed competence in reading, writing and computer skills, low consumption of fruit/vegetables and high consumption of alcoholic beverages, as well as a moderate perception of sensory health (unhealthy habits cluster), low self-perception of household economic status, low social connectedness and moderate participation in voluntary activities. This profile is associated with the four AA pillars.

**Profile IV** (people with diverse and balanced activity) was characterised by grouping subjects with a high level of competence in reading, writing and computer skills, a moderate state of health together with no need for technical or care aids, and a high frequency of participation in activities requiring moderate or vigorous physical ability, low participation in cognitive activities and moderate to high social cohesion.

**Profile V** (people with excellent active ageing conditions) brought together subjects who were relatively more heterogeneous than in the previous profiles, so the cluster category centroids are further apart (see Fig 1). This profile represents subjects with a high frequency of educational or training courses attendance and of performing cognitive activities, a very high level of financial assets or illiquid goods, as well as a high economic self-positioning of the household.

In the perceptual map there were two clusters, **H-5** (older adults with high hospital service use) and **L-7** (people with high level of competence and participation in learning courses) not clustered and distanced from the centre of the coordinate axes (0,0; 0,0), which showed little association with other AA profiles.

Profile I clustered 12.7% of participants, profile II 8.1%, profile III 32.0%, profile IV 30.4% and profile V 12.8%. The non-grouped clusters (H-5 and L-7) classified 0.8% and 3.2% of the participants, respectively.

### Relationship between active ageing profiles and sociodemographic, contextual, and quality of life conditions

**Fig 2:**
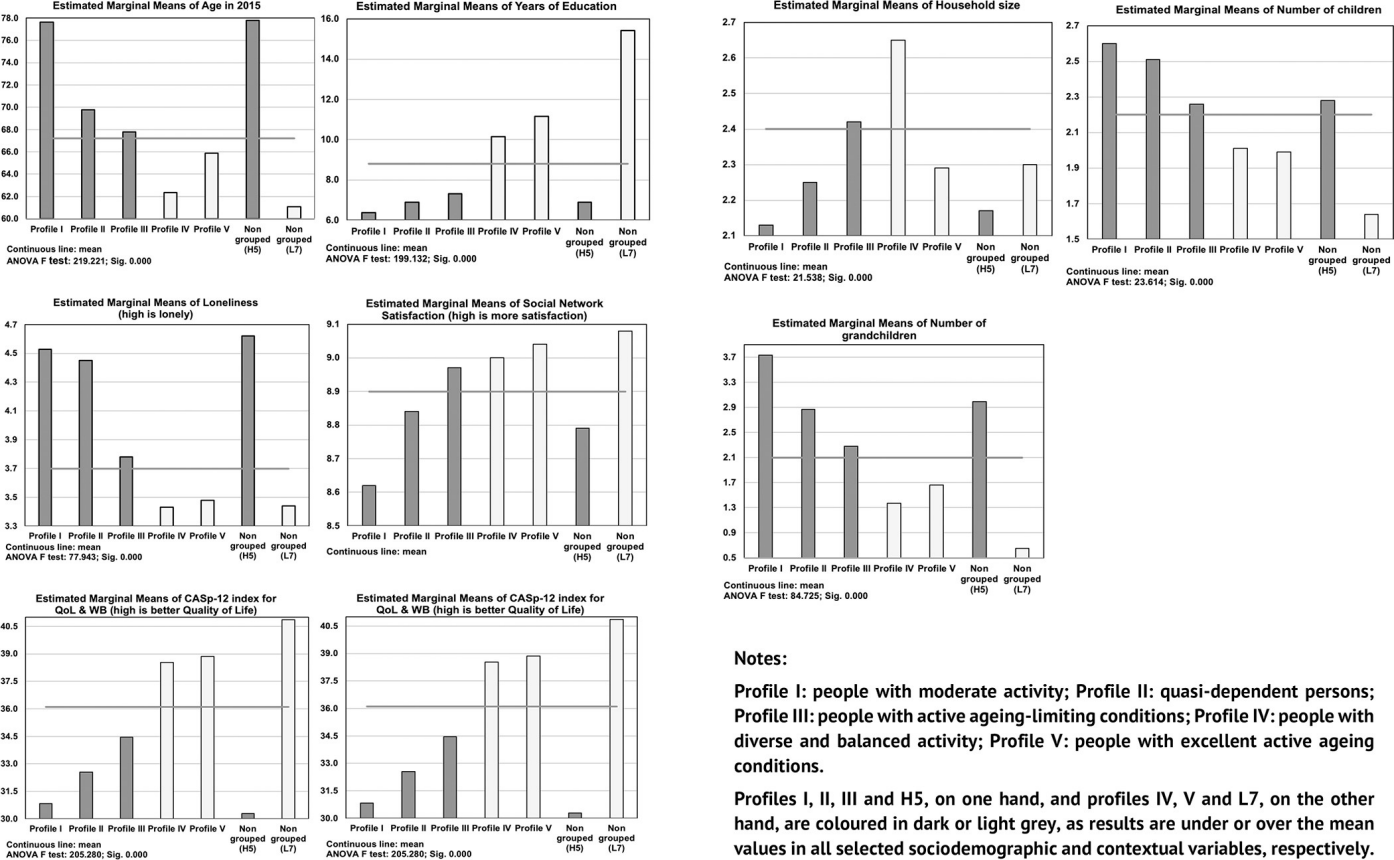
Active ageing profiles according to personal and contextual characteristics.

The results indicated a statistically significant association with all the variables analysed, and showed two behaviours depending on whether the mean values of the AA profiles were above or below the overall mean value (Fig 2) (see also the model and key results in S1 Fig). Thus, subjects in **profiles I, II and III**, as well as **cluster H-5**, were characterised by being older than average, having been in the education system for fewer years (well below the average of 8.8 years), showing a higher than average level of perceived loneliness, and lower satisfaction with their social network (except profile III) and life in general, as well as lower quality of life and subjective well-being. In home and family terms, these subjects live in small households (except profile III), and reported having an above-average number of children and grandchildren (2.2 and 2.1, respectively).

At the other end of the scale were **profiles IV and V** together with **cluster L-7.** These persons were younger (below average, 67.2 years), stayed in the educational system for more than 8.8 years on average (cluster L-7 being an extreme case as it almost doubled this average value), did not report perceiving loneliness, and achieved a higher level of satisfaction with the social network and with life as a whole, as well as a higher quality of life and subjective wellbeing.

Table 3 shows the comparison of means between the profiles, where values not sharing the same subscript (a, b, c, d, e) are significantly different. Consequently, age-related differences were observed between all AA profiles, except for cluster **H-5**, which showed no statistically significant differences with **profile I** (they share the same subscript), and cluster **L-7**, which also showed no differences with **profile IV**.

**Table 3:** Active ageing profiles according to personal and contextual characteristics (t-test with post-hoc Bonferroni correction for means comparisons).

	Profiles of Active Ageing (% of cases)							
Personal and contextual variables (mean values)	Profile I (12.7%)	Profile II (8.1%)	Profile III (32.0%)	Profile IV (30.4%)	Profile V (12.8%)	Not grouped (H-5) (0.8%)	Not grouped (L-7) (3.2%)	Mean
Age in 2015	**77.6a**	**69.8b**	**67.8c**	62.4d	65.9e	**77.8a**	61.1d	67.2
Years of education	6.4a	6.9a,b	7.3b	**10.1c**	**11.2d**	6.9a,b	**15.4e**	8.8
Loneliness (short version of R-UCLA Loneliness Scale) (high is lonely)	**4.5a**	**4.5a**	**3.8b**	3.4c	3.5c	**4.6a**	3.4c	3.7
Social network satisfaction (high is more satisfaction)	8.6a	8.8a,b	**9.0b**	**9.0b,c**	**9.0b,d**	8.8a,b	**9.1b,e**	8.9
Life satisfaction (high is more satisfaction)	7.1a	6.6b	7.3c	**7.9d**	**8.0d**	7.1a,b,c	8.5e	7.5
CASP-12 index for Quality of Life and Wellbeing (high is better QoL)	30.8a	32.6b	34.5c	**38.5d**	**38.9d**	30.3a,b	**40.9e**	36.1
Household size	2.1a	2.3a,b	**2.4b**	**2.7c**	2.3a,b	2.2a,b,c	2.3a,b	2.4
Number of children	**2.6a**	**2.5a**	**2.3b**	2.0c	2.0c,d	**2.3a,b,c,d**	1.6d	2.2
Number of grandchildren	**3.7a**	**2.9b**	**2.3c**	1.4d	1.7d	**3.0a,b,c**	0.7e	2.1

Notes: Values in the same row and subtable not sharing the same subscript (a, b, c, d, e) are significantly different at p<0.05 in the two-sided test of equality for column means. Cells with no subscript are not included in the test. Tests assume equal variances (1).

(1) Tests are adjusted for all pairwise comparisons within a row of each innermost subtable using the Bonferroni correction.

In bold, values above the mean of the total population. In shadow, not grouped clusters.

Profile I: people with moderate activity; Profile II: quasi-dependent persons; Profile III: people with active ageing-limiting conditions; Profile IV: people with diverse and balanced activity; Profile V: people with excellent active ageing conditions

Two scenarios were also observed in terms of categorical variables (Table 4): on the one hand, subjects in **profiles I, II and III** and **cluster H-5**, and, on the other, **profiles IV and V** and **cluster L-7**, although with some specific differences, such as those concerning gender, with a predominance of women in almost all profiles, of widowed people and to a lesser extent of single and divorced people. In line with the lower number of years spent in the educational system, there were more subjects from the first set of profiles (I, II, III, group H-5) in the lower levels of education, with a predominance of retirees and people with some kind of disability (permanently sick), unemployed and people engaged in housework, living in single-person households, more in single-family dwellings and even in old people’s homes (**profile I and cluster H-5**). **Profiles IV and V** and **cluster L-7** were characterised by having a high level of education, being married or divorced, still in employment, although they were also retired or engaged in housework, lived in households with their partner, or in households with their partner and with other people.

**Table 4:** Active ageing profiles according to personal and contextual categorical characteristics (contingency table; % within active ageing profile).

Variables and categories (% of cases)	Profile I (12.7%)	Profile II (8.1%)	Profile III (32.0%)	Profile IV (30.4%)	Profile V (12.8%)	Not grouped (H-5) (0.8%)	Not grouped (L-7) (3.2%)	%	
**Gender**									χ^2^ 78.780 p-value = 0.000
Male	35.9	41.2	46.0	**53.4**	45.5	31.1	38.6	46.2	
Female	**64.1**	**58.8**	**54.0**	46.6	**54.5**	**68.9**	**61.4**	53.8	
**Marital status**									χ^2^ 486.783 p-value = 0.000
Married, living with spouse	57.8	54.2	67.1	**73.1**	**71.4**	55.6	54.5	66.8	
Registered partnership	0.1	0.4	0.2	**3.5**	1.1	**2.2**	0.0	1.3	
Married, not living with spouse	**1.1**	**1.1**	1.0	0.9	**1.8**	0.0	0.0	1.1	
Never married	3.2	**10.0**	**9.6**	6.8	5.9	6.7	**15.3**	7.7	
Divorced	1.3	**6.7**	**5.4**	**7.4**	3.4	0.0	**8.0**	5.4	
Widowed	**36.4**	**27.5**	16.7	8.2	16.3	**35.6**	**22.2**	17.8	
**Education (ISCED 1997 coding)**									χ^2^ 1351.534 p-value = 0.000
0 Level 0 –Pre-Primary education	**30.6**	**18.2**	**17.4**	4.7	3.2	**22.2**	0.6	13.0	
Level 1 –Primary education or First stage of basic education	**45.0**	**50.9**	**49.3**	29.9	31.0	**46.7**	12.5	39.4	
Level 2 –Secondary (Lower) or second stage of basic education	14.3	**24.0**	20.3	**31.4**	22.9	**24.4**	2.3	23.0	
Levels 3–4 –Secondary (Upper & Post) education	5.5	1.8	8.2	**20.7**	**19.7**	4.4	**33.5**	13.4	
Levels 5–6– Tertiary (First & Second) education	4.7	5.1	4.8	**13.3**	**23.2**	2.2	**51.1**	11.2	
**Current job situation**									χ^2^ 807.523 p-value = 0.000
Retired	**45.6**	**50.4**	**41.4**	30.6	**43.1**	34.1	17.6	38.8	
Employed or self-employed	5.1	8.4	19.8	**36.4**	**27.0**	4.5	**61.4**	24.2	
Unemployed	6.6	**7.8**	**7.8**	**9.8**	1.7	0.0	**8.0**	7.4	
Permanently sick	**9.8**	**6.7**	**4.9**	0.8	3.1	**13.6**	0.0	4.1	
Homemaker	**23.2**	**24.2**	**24.5**	20.8	**23.5**	**38.6**	7.4	22.6	
Other	**9.6**	2.4	1.6	1.5	1.7	**9.1**	**5.7**	2.8	
**Household type**									χ^2^ 250.154 p-value = 0.000
Single household	**48.2**	**47.4**	**33.0**	20.5	28.9	**37.8**	**47.5**	32.3	
Household in couple & others	51.8	52.6	67.0	**79.5**	**71.1**	62.2	52.5	67.7	
**Area of building**									χ^2^ 108.021 p-value = 0.000
Rural areas	**7.6**	6.2	**8.7**	5.4	**7.5**	**11.6**	6.6	7.2	
Large/small towns	59.5	**66.5**	**65.4**	**66.8**	48.2	60.5	59.2	62.7	
A big cities/metropolitan areas	**32.9**	27.3	25.9	27.8	**44.4**	27.9	**34.2**	30.1	
**Type of building**									χ^2^ 110.210 p-value = 0.000
Farm/family house/double house	**52.7**	**51.3**	48.0	**51.1**	36.8	**50.0**	47.0	48.3	
Building with 3+ flats or high-rise	44.6	48.7	**51.5**	48.5	**63.0**	45.5	**53.0**	51.0	
Housing with services for elderly/nursing home	**2.7**	0.0	0.5	0.4	0.1	**4.5**	0.0	0.7	
**Housing tenure (owner, others)**									χ^2^ 30.090 p-value = 0.000
Owner	91.1	87.3	**92.5**	**93.5**	**92.7**	**93.2**	**98.3**	92.4	
Others (cooperative, tenant, subtenant, rent free)	**8.9**	**12.7**	7.5	6.5	7.3	6.8	1.7	7.6	

Profile I: people with moderate activity; Profile II: quasi-dependent persons; Profile III: people with active ageing-limiting conditions; Profile IV: people with diverse and balanced activity; Profile V: people with excellent active ageing conditions.

In shadow, not grouped clusters.

As for the size of the area of residence, almost two thirds of the population lived in medium-sized and large urban areas, and no clearly distinct pattern was observed between **profiles I, II and III** versus **IV and V**, as was the case with the type of residence building (3 or more storey-buildings in more than half of the cases). As for dwelling tenure status, more than 9 out of 10 older persons owned their dwelling, a status that was higher in **profiles III to V**, and **clusters H-5 and L-7**.

## Discussion

This study establishes older adult profiles according to the four AA pillars in Spain, and examines relationships between AA profiles and personal and contextual factors, well-being and quality of life. By doing so, it fills a gap in previous research: in scientific literature, the lack of **consensus on formulating the AA model** has been conditioned by discrepancies in materials and methods from a multidimensional perspective [59, 60].

As regards the first objective, consideration was given to AA’s multidimensionality based on the construction of four pillar-related indicators: Health, Lifelong Learning, Participation and Security. The aim was to overcome reductionist approaches that do not address the theoretical model or others that do not distinguish between construct criteria and determinants [64], in order to consider other analytical or methodological approaches, such as the generation of the AA index [82] the empirical validation of the AA model [18] or the study of AA and its impact on survival [24].

**Each pillar was built with multiple indicators** from the SHARE dataset [63, 65, 66]. Other authors, working with the same survey [24, 74, 83] or with other data [18], arrived at a similar selection of health domain-related indicators (diseases, dependency and physical or cognitive functioning) [84]. This paper has expanded the health-related indicators by adding others linked to the use of medical services [85], nutrition [86] and alcohol consumption [87]; all of them have been related to functionality, morbidity and/or mortality [85–87]. As regards the Participation pillar, SHARE provides information on the type of activities carried out and their frequency, usually considered by most authors [88–92]. For the Lifelong Learning pillar, consideration was given to basic skills (reading, writing or computer use) and involvement in training activities [93], while in the Security pillar, measures related mainly to financial security were considered, due to the limitation of the source. Other studies have approached Security as a pillar of manageable living conditions, such as physical security in the face of dependency [94], or the intuitive and lay understanding of older adults themselves [41].

The **analytical procedure** for handling all the information was planned and executed in successive phases, starting with the identification of patterns of relationships between variables based on PCA. The CLA conducted for each AA pillar has reflected how different older adults are as they grow older, revealing a wide variety of old age states, as the result of a process in which opportunities are used unequally within each pillar, as stated in the very definition of AA [17, 42]. Furthermore, this very powerful analytical technique is influenced by the data set used and the research strategy [95]. The resultant classification is similar to that obtained in other available reports [96]. For instance, more than half of the subjects were classified in the "moderate health" cluster of the Health pillar. According to the National Health Survey [97], 45.5% of older adults in Spain regard their health to be good or very good. Slightly more than half of the older adults were grouped in the "low competence" cluster of the Lifelong Learning pillar; according to the same report, there is a predominance of older adults with primary education and no education [96]. As for the Security pillar classification, the fact that almost 60% of the subjects are grouped in the "Self-assessed low economic status" cluster could be explained by the volume of inactive population in the sample studied (around 24% were still employed) and the effect of retirement on income [98]. This is in line, firstly, with the individuals’ own self-perception of their **financial resources** [99] and the **reported difficulties in making ends meet**, which are particularly noticeable in Eastern and Southern European countries [100], and, secondly, with the fact that they are close to the poverty line [96] which could be a limiting factor in promoting AA and enhancing the quality of life of older adults.

An optimal combination of the Health, Lifelong Learning, Participation and Security pillars will be key to achieving AA. Most authors hold this assumption and there have been attempts to build it into empirical models [101–103], yet few have succeeded in showing the interdependence between some pillars and others to show profiles of older adults ageing along diverse trajectories. This paper has demonstrated the interdependence of the pillars, giving **five main profiles**, which in turn were related to personal and contextual factors as well as to measures of well-being and quality of life.

Worth noting is that **engaging in activities** is present in all profiles except the one defined only by the Health pillar (profile II). **Health**, through the multiple measures used, either as a factor or as an outcome, is another area that is closely linked with the activity profiles. Indeed, a low perception of **health, a limited level of functioning and unhealthy habits** lead to less activity among older adults [28, 104], while more favourable health conditions encourage leisure and participation activities, such as volunteer work [33, 105]. This is probably due to the fact that Participation is a cornerstone of the AA framework [92, 106] and is a defining element, as opposed to other related terms such as successful, productive, or positive [47, 84]. Moderate or high participation is related to moderate health and economic conditions. These variables are associated in a multitude of studies and their interdependence is clearly evident [52, 107, 108]. Health and Security seem to be the necessary elements [22, 41] underpinning Participation [106]. In addition, better conditions in the Lifelong Learning pillar [15] were related to higher activity profiles.

By analysing profiles of older adults in Spain, this research has identified a wide **range of factors** that give them interpretative consistency, but which do not always match those offered in AA literature, either because they do not follow the same theoretical basis or because they do not always use the same analytical methodology. That is why the AA profiles are constructed with quantitative methods that combine independent variables, generally at the individual level, with others that express the results of the AA process or other related facts, such as quality of life or subjective well-being as outcome variables. As a consequence, it is often hard to clearly distinguish between dimensions, determinants and outcomes, because the analyses are too closely linked to the available data. However, so of the many different factors that influence older adults’ active behaviours are far more prevalent than others.

The **basic demographic variables**, age and gender, are part of the most common interpretative construct because they feature in all studies, whatever their type. **Age** plays a preferential role, yet it tends to act in two directions: firstly, by appearing in the least active groups [109] and, secondly, by influencing the reduction in the number and type of activities as the population ages [28, 52], although it is not always documented to work this way [110]. Similarly, the age variable shows a different association by type of active elderly, depending on whether the activities are carried out at home (at older ages) such as family help or home maintenance, or outside the home (at younger ages) such as volunteer work [95]. The fact is that, by including other age-related variables, this activity trajectory is also related to living without a partner, with lower economic income [104] and a decrease in personal well-being [50].

**Women’s involvement** is greater in some specific profiles, such as those involving caring for people or activities in the home, or less when it comes to profiles of people still linked to the world of work or volunteer work [95, 109–111]. In the case of Spain, the life trajectory of these post-Civil War (post-1939) generations, marks an appreciable difference in **gender roles**, although recently women seem to be more interested in carrying out ’novel’ and motivating activities, which are more rewarding and which allow them to recover a role hitherto not usually assigned to them [29]. Men of these generations behave more conservatively and are more attached to the closer and less active social community space.

The **level of education**, measured by the number of years spent in the system and the level attained, is another factor that conditions the activity profile, through general rules: a lower educational level tends to be associated with less activity [28, 110] and less rewarding or motivating activities, but of a compulsory nature in the family sphere [110]. The profiles obtained also show intergenerational educational level-related gains. For instance, profiles IV and V are more defined by the Lifelong Learning, with younger ages and a higher level of education. Different studies point to the country’s older adults having higher levels of education, making it possible to reduce the gender gap in old age [112].

From a life course perspective, the population studied includes people who are old enough to be retired from work or who are carrying out household tasks, as the main **activity**-related groups. Both can guide their transition into retirement through a variety of possibilities [113], from those requiring remuneration to those undertaken on a voluntary basis [114] or to maintain intergenerational care relationships [115]. However, activity-relatedness is not a factor in many AA studies, probably because of the limited ability to discriminate if the vast majority of the population is already retired or because it is mediated by other variables such as age [116]. However, this factor becomes relevant when analysed together with many others to relate AA to quality of life [48, 56].

Another way of influencing activity is through concomitant variables, such as **level of income**, so that education and economy are associated in determining activity profiles [105], or **marital status** to indicate that people who live alone and have a low level of education behave in a similar way [95]. Precisely, beyond marital status, the form of **cohabitation, the size of the household and having children and grandchildren** are relevant variables in the differential characterisation of activity profiles. The key could be found in whether there are children (or even grandchildren) in the household, or within the family network but living outside the household, in more or less close environments and with more or less frequent contacts in an ascending familialism or supportive-at-distance typology [117]. In the first case, a larger household size and reporting having few children and grandchildren is consistent with a profile of younger people, and, in general, men, people living in a couple and with others, possibly children yet to be emancipated, who maintain a diverse and balanced activity (profile IV). Something similar happens with profile V, but in this case they would be women. At the same time, having more children and grandchildren corresponds to low activity profiles (profiles I and II): people living alone, in smaller households, older and, above all, women. **Yet having more children and grandchildren could also tend to lead to activities in the home or family care environment** that compete with other leisure and participation activities for the person’s available time, in order to reconcile tasks of different types and nature [110]. The latter could be the case of the profile of limiting conditions for AA (profile III), which is observed among not very old women who say that they have more children and grandchildren both inside and outside the home, and which would also correspond to a descending familialism typology and activity based on intergenerational family solidarity provided by older women [118]. In any case, profiles I, II and III show higher reported loneliness, compared to lower scores for profiles IV and V, which would be related not so much to the size of the family network but rather to other factors such as increasing age and changes and lost in marital status, income, self-rate health, cognitive functioning and depression [119, 120], aspects that are also related to limiting conditions for AA and maintaining a good quality of life [121].

Personal motivation (or a lack thereof) as well as personal rewards (**life satisfaction**) and social rewards (**social networks, avoidance of loneliness**) also contribute to understanding the active behaviour of older adults [106]. It has been found that having a higher number of people in one’s social network is associated with higher levels of activity, while a less dense network is associated with lower activity, although perceived support may act in the opposite direction [28, 104]. On the other hand, the importance of the social and community environment in which the activities are carried out must be assessed as a mechanism for reinforcing them [106].

As regards other **contextual conditions**, older adults tend to reside in **cities**, especially medium-sized ones [122], which mirrors the process of urbanisation and demographic ageing [123–125]. In this study, no homogeneous pattern has been observed according to the two large profile groups, such that both profile I and V subjects reside in large urban and metropolitan areas, while the remaining ones do so in smaller cities. In any case, the trend towards urbanisation has led to the development of a specific city friendliness programme in order to optimise the living conditions and quality of life of older adults [30, 126].

With respect to the residential environment, **home ownership** is the most significant regime in Spain compared to other neighbouring countries [127], and among the older population it reaches higher proportions in line with their age and the time they have had to acquire it [128]. The results show that people with the worst AA conditions (profiles I, II and III, located in the low scores of dimension 1 of the perceptual map) showed slightly lower percentages of ownership compared to the profiles of better positioned subjects, in accordance with their greater purchasing power. In relation to the **type of residence dwelling**, two situations were observed; on the one hand, older adults with a moderate active profile, living to a greater extent on a farm or in family housing, in line with their location in smaller residential areas, and, on the other hand, the profiles of younger people with better AA conditions, living in housing in block buildings in line with their settlement in large cities and metropolitan areas. In Spain, part of the older population faces the problems of an ageing housing stock characterised by a lower level of amenities (lifts, heating, air conditioning) and the need for renovations, which worsen their isolation, hinder the desire to grow old at home with autonomy and independence, and jeopardise the promotion of AA [128, 129].

Other factors may also influence the level of activity, but their effects are not differentiated because they are incorporated into the more general variables. Something very similar happens when we try to measure the impact of carrying out more or less activities of one type or another on **personal well-being, quality of life or satisfaction with it**. These are very general social and multidimensional constructs, in which it is not the influence of all their conditioning factors is not easily identifiable, and their effects may be contradictory depending on the research design and the data used [50, 130]. **The relationship between AA and personal well-being** (including life satisfaction, quality of life, satisfaction with social networks, absence of perceived loneliness) has been highlighted in the profiles of older adults who are more competent and with better personal and contextual conditions to have a high level of activity, in line with the high association of these constructs [19, 92].

Constructing an ageing model based on a broad set of variables, in order to identify profiles of older adults with different degrees of activity, is a significantly increasing trend in the literature, and one that uses a methodology based on individual data with multidimensional variables: some that measure different activities, the "process" variables [130] while others measure the person’s situation and which the AA model accepts as determining factors. Yet the multidimensional approach is also entails far more complex, as this paper has shown with regard to the construction of AA profiles. The use of quantitative data, from SHARE or other European and North American databases, has highlighted the potential of this classification strategy, both in terms of the activities analysed and the determinants that serve to explain the types of activity and/or profiles of older adults, measured from different perspectives (individual, countries) and supported by different theories [28, 38, 50, 52, 92, 109, 110, 131–133]. This paper has also revealed a far from negligible diversity of results influenced by the population samples and the variables selected and available for analysis [104, 133, 134]. Furthermore, one must not lose sight of the interpretative capacity of using qualitative information in the study of AA profiles [29]. The tendency, however, is that the WHO AA model is not usually considered as the reference to be followed in studies on activity profiles and older adults, and when it is, not all dimensions and determinants are covered [133]. It is much more common to use various unidimensional, multidimensional or behavioural models, according to Boudiny [135], based on successful, healthy or productive ageing theories, using specific sources that do not make it easy to standardise results. The sample of studies cited above are good evidence of this.

### Limitations and future lines

It must be noted that this research was subject to certain limitations. The first stems from the difficulty of finding data on AA [136]. This study used a database, the SHARE project, which is characterised by its rich multidimensional design, and the fact that it studies a large number of countries, thus permitting cross-sectional and longitudinal comparative studies. However, this survey is not designed to specifically survey AA. So, from a thematic approach, this dataset does not offer all the information defined in the AA paradigm [17, 42]. In this regard, an unequal number of variables have been used per pillar, which also conditions the different number of variables involved in its construction, on the one hand, and a possible bias in the results, on the other.

The larger number of indicators available in the SHARE survey matches the areas of greatest scientific development within AA, namely Health and Participation, with a lower presence of questions related to the Lifelong Learning and Security pillars, despite their proven relevance in positive ageing trajectories [14, 103, 137].

As regards the variables selected in the Participation pillar, almost 30% of the participants had missing values. Therefore, during the PCA of this pillar, these values were replaced by the mean of the variable. This may have influenced the results obtained for this pillar in the first CLA run, as almost 95% of the cases were grouped around a single cluster. This was the reason why, for this set of variables, the initial centroids were user-defined.

Another limitation of the study is the database date, 2015 year. SHARE-ERIC Consortium carried out two subsequent waves in 2017 (wave 7) and 2019 (wave 8). Wave 7 lacked the appropriate information about social networks as a relevant domain in AA. The wave 8 did not have the data available at the time of execution of our research because the field work was interrupted by the COVID-19 pandemic [138]. This paper, based on wave 6, showed the generation of active ageing profiles, and we assume that the changes in the two following waves would be smaller due to the short difference in dates. However, the COVID-19 pandemic outbreak could have altered older adults behaviors and quality of life due to i) the consequences of the impact of the disease, and ii) the measures imposed for preventing the spread of the coronavirus, showed by other research [139–145]. In this sense, the SHARE-ERIC designed and developed the SHARE—COVID19 survey (1 and 2 waves) (http://www.share-project.org/share-covid19.html) with the objective of identifying the lockdown effects over older-adult population. This is a future line of research to examine the possible impact of the pandemic on active ageing behaviors.

Despite these limitations, the research also has certain **strengths**, including the methodological design to address the study of a large dataset of different types of data. Consequently, the successive analytical procedure phases have been expressly planned and executed for the proposed objectives.

In the multiple and diverse AA studies, there is room for **future and novel developments** stemming from their conceptualisation and progress and from the aforementioned limitations to achieve more precise diagnoses. Some of the possible improvements in these studies should come from the need to establish comparative frameworks between countries, differentiated by their social, cultural and political model, thus overcoming the reductionism imposed by research anchored in, for example, developed countries. Although this is an increasingly widespread trend, there are two other areas that would require more attention, such as cross-referencing and further triangulation studies. Both would stem from longitudinal type analyses, albeit constrained by the availability of adequate data, and the use of combined quantitative-qualitative methodologies, which would make it easier to compare the two visions and provide deeper insight into the views and experiences of older adults.

Finally, there is another area for improvement in AA research, derived from the use of pre-post methodology, which enables psychosocial and environmental interventions to first assess and then improve the behaviour of older adults. These interventions also can contribute to the development of actions aimed at promoting AA at an individual and collective level, by encouraging healthy lifestyles, developing active and passive preventive safety strategies in old age, and promoting active participation and continuous learning [30, 146]. In turn, environmental interventions, based on designs and adjustments of the residential and community environment, such as environmental adaptations, relevant assistive technologies, and environmental and behavioral safety strategies, can favour changes in behaviors and promote better environmental adaptation of old people, encouraging outdoor activities, participation and social relationships, and reducing the risk of social isolation and loneliness [147, 148]. This knowledge would underpin the application of public policies aimed at promoting AA as a mechanism for consolidating quality of life in the ageing process.

## Conclusions

This research has revealed the presence of various profiles of older adults according to their levels of AA in Spain. Following the pillars of the seminal WHO model and its subsequent complementation and applying various analytical statistical techniques, five profiles of people have been obtained: with moderate activity, quasi-dependents, with limiting AA conditions, with diverse and balanced activity, and with excellent AA conditions. The first three profiles accounted for more than half of the population, their main features being their higher average age, lower level of education, being retired, living in small households but having had more children and grandchildren, showing a greater perception of loneliness and lower quality of life. On the other hand, profile IV and V subjects were the mirror image of the previous profiles.

With the results obtained, the older adult subjects can be classified into profiles, which could serve as a basis for establishing intervention priorities, although this is not the object of this study. However, the main priority would be to address the foundations for better living conditions in old age throughout the life cycle, from educational stages, working age, retirement age, or other stages with specific needs. As a process, ageing reflects a person’s previous way of life [10] and, as a society, possible differences in the life course will lead to social inequalities [149], which are at the origin of a different level of AA. As the older adults group grows with the arrival of generations with better living conditions, this age group is likely to achieve better AA profiles in the near future.

## Supporting information

S1 Table Selected variables according to active ageing pillars, and personal and contextual information. (PDF)

S2 Table Factor analysis by principal component analysis extraction method. (PDF)

S1 Fig Active ageing profiles model and key results. (PDF)

## References

[1] Christensen K, Doblhammer G, Rau R, Vaupel JW. Ageing populations: the challenges ahead. The Lancet. 2009;374(9696):1196–208. doi: 10.1016/S0140-6736(09)61460-4 19801098 PMC2810516

[2] UN- United Nations, Department of Economic and Social Affairs, Population Division. World Population Ageing 2019 (ST/ESA/SER.A/444). New York: United Nations, Department of Economic and Social Affairs, Population Division; 2020. Available from: https://www.un.org/en/development/desa/population/publications/pdf/ageing/WorldPopulationAgeing2019-Report.pdf

[3] Baltes PB, Smith J. New frontiers in the future of aging: from successful aging of the young old to the dilemmas of the fourth age. Gerontology. 2003;49(2):123–35. doi: 10.1159/000067946 12574672

[4] Rowe JW, Kahn RL. Successful aging. The Gerontologist. 1997;37(4):433–40. doi: 10.1093/geront/37.4.433 9279031

[5] Fernández-Ballesteros R, Sánchez-Izquierdo M, Santacreu M. Active Aging and Quality of Life. In: Rojo-Pérez F, Fernández-Mayoralas G, editors. Handbook of Active Ageing and Quality of Life From Concepts to Applications. Cham: Springer, International Handbooks of Quality-of-Life series; 2021. p. 15–42. doi: 10.1007/978-3-030-58031-5_2

[6] Costa-Font J, Wittenberg R, Patxot C, Comas-Herrera A, Gori C, di Maio A, et al. Projecting Long-Term Care Expenditure in Four European Union Member States: The Influence of Demographic Scenarios. Social Indicators Research. 2008;86(2):303–21. doi: 10.1007/s11205-007-9140-4

[7] Akosile CO, Mgbeojedo UG, Maruf FA, Okoye EC, Umeonwuka IC, Ogunniyi A. Depression, functional disability and quality of life among Nigerian older adults: Prevalences and relationships. Archives of Gerontology and Geriatrics. 2018;74:39–43. doi: 10.1016/j.archger.2017.08.011 28954240

[8] Naveiro-Rilo J-C, Diez-Juárez D, Flores-Zurutuza M-L, Javierre-Pérez P, Alberte-Pérez C, Molina-Mazo R. La calidad de vida en ancianos polimedicados con multimorbilidad. Revista Española de Geriatría y Gerontología. 2014;49(4):158–64. doi: 10.1016/j.regg.2013.10.004 24529640

[9] Zaidi A, Gasior K, Zolyomi E, Schmidt A, Rodrigues R, Marin B. Measuring active and healthy ageing in Europe. Journal of European Social Policy. 2017;27(2):138–57. doi: 10.1177/0958928716676550

[10] Kalache A, Voelcher I, Louvison M. Active Aging and the Longevity Revolution. In: Rojo-Pérez F, Fernández-Mayoralas G, editors. Handbook of Active Ageing and Quality of Life From Concepts to Applications. Cham: Springer, International Handbooks of Quality-of-Life series; 2021. p. 43–62. doi: 10.1007/978-3-030-58031-5_3

[11] Walker A, Maltby T. Active ageing: A strategic policy solution to demographic ageing in the European Union. International Journal of Social Welfare. 2012;21(Suppl. 1):S117–S30. doi: 10.1111/j.1468-2397.2012.00871.x

[12] Rojo-Pérez F, Fernández-Mayoralas G, Rodríguez-Rodríguez V. Active Ageing and Quality of Life: A Systematized Literature Review. In: Rojo-Pérez F, Fernández-Mayoralas G, editors. Handbook of Active Ageing and Quality of Life From Concepts to Applications. Cham: Springer, International Handbooks of Quality-of-Life series; 2021. p. 63–96. doi: 10.1007/978-3-030-58031-5_4

[13] Rojo-Pérez F, Gallardo-Peralta L, Fernández-Mayoralas G, Rodríguez-Rodríguez V, Montes de Oca Zavala V, Prieto-Flores ME, et al. Envejecimiento activo y buen envejecer en Iberoamérica. Una revisión bibliográfica. In: Fernández-Mayoralas G, Rojo-Pérez F, editors. Envejecimiento Activo, Calidad de Vida y Género Las miradas académica, institucional y social. Valencia: Tirant lo Blanch; 2021. p. 175–211. Available from: https://editorial.tirant.com/co/libro/envejecimiento-activo-calidad-de-vida-y-genero-9788418329142

[14] Molina MÁ, Schettini R. Lifelong Learning and Quality of Life. In: Rojo-Pérez F, Fernández-Mayoralas G, editors. Handbook of Active Ageing and Quality of Life From Concepts to Applications. Cham: Springer, International Handbooks of Quality-of-Life series; 2021. p. 111–9. doi: 10.1007/978-3-030-58031-5_6

[15] Schettini R, Molina-Martínez MÁ, Gallardo Peralta LP. Formación continua en el proceso de envejecimiento desde una perspectiva popular. In: Fernández-Mayoralas G, Rojo-Pérez F, editors. Envejecimiento Activo, Calidad de Vida y Género Las miradas académica, institucional y social. Valencia: Tirant lo Blanch; 2021. p. 307–26. Available from: https://editorial.tirant.com/co/libro/envejecimiento-activo-calidad-de-vida-y-genero-9788418329142

[16] Martín Palomo MT, Muñoz Terrón JM. Tecnologías de la Información y de la Comunicación en el envejecimiento activo. In: Fernández-Mayoralas G, Rojo-Pérez F, editors. Envejecimiento Activo, Calidad de Vida y Género Las miradas académica, institucional y social. Valencia: Tirant lo Blanch; 2021. p. 409–528. Available from: https://editorial.tirant.com/co/libro/envejecimiento-activo-calidad-de-vida-y-genero-9788418329142

[17] WHO- World Health Organization. Active Ageing: A Policy Framework. Geneva: World Health Organization; 2002. 1–60 p. Available from: http://apps.who.int/iris/bitstream/10665/67215/1/WHO_NMH_NPH_02.8.pdf 12040973

[18] Paúl C, Ribeiro O, Teixeira L. Active ageing: An empirical approach to the WHO model. Current Gerontology and Geriatrics Research. 2012(382972):1–10. doi: 10.1155/2012/382972 23193396 PMC3501803

[19] Rojo-Pérez F, Fernández-Mayoralas G. Comprensión y autovaloración de la Calidad de Vida como medida de resultado del Envejecimiento Activo. In: Fernández-Mayoralas G, Rojo-Pérez F, editors. Envejecimiento Activo, Calidad de Vida y Género Las miradas académica, institucional y social. Valencia: Tirant lo Blanch; 2021. p. 743–801. Available from: https://editorial.tirant.com/co/libro/envejecimiento-activo-calidad-de-vida-y-genero-9788418329142

[20] Win HH, Nyunt TW, Lwin KT, Zin PE, Nozaki I, Bo TZ, et al. Cohort profile: healthy and active ageing in Myanmar (JAGES in Myanmar 2018): a prospective population-based cohort study of the long-term care risks and health status of older adults in Myanmar. BMJ Open. 2020;10(10):e042877. doi: 10.1136/bmjopen-2020-042877 33130574 PMC7783620

[21] Bosch-Farré C, Garre-Olmo J, Bonmatí-Tomàs A, Malagón-Aguilera M-C, Gelabert-Vilella S, Fuentes-Pumarola C, et al. Prevalence and related factors of Active and Healthy Ageing in Europe according to two models: Results from the Survey of Health, Ageing and Retirement in Europe (SHARE). PLOS One. 2018;13(10):1–19. doi: 10.1371/journal.pone.0206353 30372472 PMC6205806

[22] Rodríguez-Blázquez C, Martin S, Forjaz MJ. Factores determinantes de la salud en el envejecimiento activo. In: Fernández-Mayoralas G, Rojo-Pérez F, editors. Envejecimiento Activo, Calidad de Vida y Género Las miradas académica, institucional y social. Valencia: Tirant lo Blanch; 2021. p. 213–29. Available from: https://editorial.tirant.com/co/libro/envejecimiento-activo-calidad-de-vida-y-genero-9788418329142

[23] Fass E, Schlesinger T. The Relation of Physical Activity and Self-Rated Health in Older Age—Cross Country Analysis Results from SHARE. Journal of Population Ageing. 2020;13(3):347–64. doi: 10.1007/s12062-019-09242-w

[24] Hijas-Gómez AI, Ayala A, Rodríguez-García MP, Rodríguez-Blázquez C, Rodríguez-Rodríguez V, Rojo-Pérez F, et al. The WHO Active Ageing Pillars and its association with survival: findings from a population-based study in Spain. Archives of Gerontology and Geriatrics. 2020;90(104114):1–15. doi: 10.1016/j.archger.2020.104114 32526561

[25] Litwin H, Schwartz E, Damri N. Cognitively Stimulating Leisure Activity and Subsequent Cognitive Function: A SHARE-based Analysis. The Gerontologist. 2017;57(1): 940–8. doi: 10.1093/geront/gnw084 27117305 PMC5881687

[26] Sirven N, Debrand T. La Participation Sociale des Personnes Âgées en Europe. Instrument du «Bien Vieillir » ou Facteur d’Inégalités Sociales de Santé? [Social participation of elderly people in Europe: An instrument of "healthy ageing" or a factor in the social inequality of health?]. Retraite et Société. 2013;65(2):59–80. doi: 10.3917/rs.065.0059

[27] Fernández-Mayoralas G, Rojo-Pérez F, Martínez-Martín P, Prieto-Flores M-E, Rodríguez-Blázquez C, Martín-García S, et al. Active ageing and quality of life: factors associated with participation in leisure activities among institutionalized older adults, with and without dementia. Aging & Mental Health. 2015;19(11):1031–41. doi: 10.1080/13607863.2014.996734 25584744

[28] Morrow-Howell N, Putnam M, Lee YS, Greenfield JC, Inoue M, Chen H. An Investigation of Activity Profiles of Older Adults. The Journals of Gerontology, Series B: Psychological Sciences and Social Sciences. 2014;69(5):809–21. doi: 10.1093/geronb/gbu002 24526690 PMC4189653

[29] Rodríguez-Rodríguez V, Rojo-Pérez F, Fernández-Mayoralas G, Prieto-Flores M-E. ¿Cómo interpretan el envejecimiento activo las personas mayores en España? Evidencias desde una perspectiva no profesional. Aula Abierta. 2018;47(1):67–78. doi: 10.17811/rifie.47.1.2018.67–78

[30] Sánchez-González D, Rojo-Pérez F, Rodríguez-Rodríguez V, Fernández-Mayoralas G. Environmental and Psychosocial Interventions in Age-Friendly Communities and Active Ageing: A Systematic Review. International Journal of Environmental Research and Public Health. 2020;17(22):8305. doi: 10.3390/ijerph17228305 33182710 PMC7696667

[31] Haski-Leventhal D. Elderly Volunteering and Well-Being: A Cross-European Comparison Based on SHARE Data. VOLUNTAS: International Journal of Voluntary and Nonprofit Organizations. 2009;20(4):388–404. doi: 10.1007/s11266-009-9096-x

[32] Morawski L, Okulicz-Kozaryn A, Strzelecka M. Elderly Volunteering in Europe: The Relationship Between Volunteering and Quality of Life Depends on Volunteering Rates. VOLUNTAS: International Journal of Voluntary and Nonprofit Organizations. 2020 first online;. doi: 10.1007/s11266-020-00267-w

[33] Papa R, Cutuli G, Principi A, Scherer S. Health and Volunteering in Europe: A Longitudinal Study. Research on Aging. 2019;41(7):670–96. doi: 10.1177/0164027519834939 30845894

[34] Potocnik K, Sonnentag S. A longitudinal study of well-being in older workers and retirees: The role of engaging in different types of activities. Journal of Occupational and Organizational Psychology. 2013;86(4):497–521. doi: 10.1111/joop.12003

[35] Principi A, Galenkamp H, Papa R, Socci M, Suanet B, Schmidt A, et al. Do predictors of volunteering in older age differ by health status? European Journal of Ageing. 2016;13(2):91–102. doi: 10.1007/s10433-016-0377-0 28804374 PMC5550605

[36] Serrat R, Scharf T, Villar F, Gómez C. Fifty-Five Years of Research Into Older People’s Civic Participation: Recent Trends, Future Directions. The Gerontologist. 2020;60(1):e38–e51. doi: 10.1093/geront/gnz021 30889249 PMC12774630

[37] Litwin H, Stoeckel K. Engagement and social capital as elements of active ageing: an analysis of older europeans. Socilogia e Politiche Sociali. 2014;17(3):9–31. doi: 10.3280/SP2014-003002

[38] Rossi G, Boccacin L, Bramanti D, Meda SG. Active ageing: Intergenerational relationships and social generativity. In: Riva G, Ajmone Marsan P, Grassi C, editors. Active Ageing and Healthy Living: A Human Centered Approach in Research and Innovation as Source of Quality of Life. Amsterdam: IOS Press. Series Studies in Health and Technology, vol. 203; 2014. p. 57–68. doi: 10.3233/978-1-61499-425-1-57 26630512

[39] Di Gessa G, Grundy E. The relationship between active ageing and health using longitudinal data from Denmark, France, Italy and England. Journal of Epidemiology and Community Health. 2014;68(3):261–7. doi: 10.1136/jech-2013-202820 24272919

[40] Wahrendorf M, Siegrist J. Are changes in productive activities of older people associated with changes in their well-being? Results of a longitudinal European study. European Journal of Ageing. 2010;7(2):59–68. doi: 10.1007/s10433-010-0154-4 28798618 PMC5547334

[41] Fernández-Mayoralas G, Rojo-Pérez F, Rodríguez-Rodríguez V, Sánchez-Román M, Schettini R, Rodríguez-Blázquez C, et al. Marco teórico y estudio de diseño e implementación de investigación cualitativa en Envejecimiento Activo, Calidad de Vida y Género. In: Fernández-Mayoralas G, Rojo-Pérez F, editors. Envejecimiento Activo, Calidad de Vida y Género Las miradas académica, institucional y social. Valencia: Tirant lo Blanch; 2021. p. 48–126. Available from: https://editorial.tirant.com/co/libro/envejecimiento-activo-calidad-de-vida-y-genero-9788418329142

[42] ILC-BR- International Longevity Centre Brazil. Active Ageing: A Policy Framework in Response to the Longevity Revolution. Rio de Janeiro: International Longevity Centre Brazil; 2015. 116 p. Available from: https://ilcbrazil.org.br/wp-content/uploads/2020/07/FINAL-executive-summary-04-v1.1.pdf

[43] Molina MÁ, Cañadas-Reche JL, Serrano-del-Rosal R. Social Participation of the Elders in Europe: The Influence of Individual and Contextual Variables. Ageing International. 2018;43(2):190–206. doi: 10.1007/s12126-017-9300-z

[44] Sánchez-Román M, Fernández-Mayoralas G. Género y envejecimiento activo: la visión de los informantes clave en instituciones, equipos profesionales y personas mayores usuarias de servicios. In: Fernández-Mayoralas G, Rojo-Pérez F, editors. Envejecimiento Activo, Calidad de Vida y Género Las miradas académica, institucional y social. Valencia: Tirant lo Blanch; 2021. p. 585–616. Available from: https://editorial.tirant.com/co/libro/envejecimiento-activo-calidad-de-vida-y-genero-9788418329142

[45] Steinmayr D, Weichselbaumer D, Winter-Ebmer R. Gender Differences in Active Ageing: Findings from a New Individual-Level Index for European Countries. Social Indicators Research. 2020;151(2):691–721. doi: 10.1007/s11205-020-02380-1

[46] Hank K, Erlinghagen M. Dynamics of volunteering in older Europeans. The Gerontologist. 2010;50(2):170–8. doi: 10.1093/geront/gnp122 19666783 PMC2838410

[47] Molina-Martínez MA, Schettini R, Fernández-Fernández V, Gallardo-Peralta L. El papel de las variables psicológicas en el envejecimiento activo. Perspectiva no académica. In: Fernández-Mayoralas G, Rojo-Pérez F, editors. Envejecimiento Activo, Calidad de Vida y Género Las miradas académica, institucional y social. Valencia: Tirant lo Blanch; 2021. p. 473–95. Available from: https://editorial.tirant.com/co/libro/envejecimiento-activo-calidad-de-vida-y-genero-9788418329142

[48] Rojo-Pérez F, Fernández-Mayoralas G, Rodríguez-Rodríguez V, Lardiés-Bosque R, Prieto-Flores ME, Gallardo-Peralta LP, et al. Contextos residenciales, envejecimiento activo y calidad de vida. Un análisis a microescala en España. In: Sempere Souvannavong JD, Cortés Samper C, Cutillas Orgilés E, Valero Escandell JR, editors. Población y territorio España tras la crisis de 2008. Granada: COMARES; 2020. p. 191–208. Available from: http://rua.ua.es/dspace/handle/10045/115389

[49] Lardiés Bosque R. Sociedad, instituciones y políticas: factores para envejecer activamente y con calidad de vida en España. In: Fernández-Mayoralas G, Rojo-Pérez F, editors. Envejecimiento Activo, Calidad de Vida y Género Las miradas académica, institucional y social. Valencia: Tirant lo Blanch; 2021. p. 397–438. Available from: https://editorial.tirant.com/co/libro/envejecimiento-activo-calidad-de-vida-y-genero-9788418329142

[50] Ramia I, Voicu M. Life Satisfaction and Happiness Among Older Europeans: The Role of Active Ageing. Social Indicators Research. 2022;160:667–87. doi: 10.1007/s11205-020-02424-6

[51] Erlinghagen M, Hank K. The participation of older Europeans in volunteer work. Ageing & Society. 2006;26(4):567–84. doi: 10.1017/S0144686X06004818

[52] Arpino B, Bordone V. Active Ageing Typologies: A Latent Class Analysis of the Older Europeans. In: Zaidi A, Harper S, Howse K, Lamura G, Perek-Białas J, editors. Building Evidence for Active Ageing Policies: Active Ageing Index and its Potential. Singapore: Springer Singapore; 2018. p. 295–311. doi: 10.1007/978-981-10-6017-5_14

[53] Au DWH, Woo J, Zaidi A. Extending the Active Ageing Index to Hong Kong Using a Mixed-Method Approach: Feasibility and Initial Results. Journal of Population Ageing. 2021;14(1):53–68. doi: 10.1007/s12062-020-09275-6

[54] Barslund M, Von Werder M, Zaidi A. Inequality in active ageing: evidence from a new individual-level index for European countries. Ageing & Society. 2019;39(3):541–67. doi: 10.1017/S0144686X17001052

[55] Rodríguez-Rodríguez V, Rojo-Pérez F, Fernández-Mayoralas G, Morillo R, Forjaz MJ, Prieto-Flores ME. Active Ageing Index: Application to Spanish Regions. Journal of Population Ageing. 2017;10(1):25–40. doi: 10.1007/s12062-016-9171-1

[56] Rojo-Pérez F, Fernández-Mayoralas G, Rodríguez-Rodríguez V. Active Ageing and Personal Wellbeing Among Older Adults in Spain. In: Maggino F, editor. Encyclopedia of Quality of Life and Well-Being Research. Cham: Springer International Publishing; 2020. p. 1–10. doi: 10.1007/978-3-319-69909-7_4001–2

[57] Varlamova M, Sinyavskaya O. Active Ageing Index in Russia—Identifying Determinants for Inequality. Journal of Population Ageing. 2020;14(1):69–90. doi: 10.1007/s12062-020-09277-4

[58] Um J, Zaidi A, Parry J, Xiong Q. Capturing gendered aspects of active aging in China: Insights drawn from the Active Aging Index in comparison with EU countries. Asian Social Work and Policy Review. 2020;15(1):47–59. doi: 10.1111/aswp.12218

[59] Álvarez-García J, Durán-Sánchez A, Del Río-Rama MDlC, García-Vélez DF. Active Ageing: Mapping of Scientific Coverage. International Journal of Environmental Research and Public Health. 2018;15:1–21. doi: 10.3390/ijerph15122727 30513943 PMC6313563

[60] Naah FL, Njong AM, Kimengsi JN. Determinants of active and healthy ageing in sub-saharan africa: Evidence from Cameroon. International Journal of Environmental Research and Public Health. 2020;17(9):3038. doi: 10.3390/ijerph17093038 32349334 PMC7246554

[61] Thanakwang K, Isaramalai S, Hatthakit U. Development and psychometric testing of the active aging scale for Thai adults. Clinical Interventions in Aging. 2014;9:1211–21. doi: 10.2147/CIA.S66069 25092971 PMC4116362

[62] Borsch-Supan A, Hank K, Jurges H, Schroder M. Introduction: empirical research on health, ageing and retirement in Europe. Journal of European Social Policy. 2009;19(4):293–300. doi: 10.1177/1350506809341510

[63] Börsch-Supan A, Brandt M, Hunkler C, Kneip T, Korbmacher J, Malter F, et al. Data Resource Profile: The Survey of Health, Ageing and Retirement in Europe (SHARE). International Journal of Epidemiology. 2013;42(1):992–1001. doi: 10.1093/ije/dyt088 23778574 PMC3780997

[64] Fernández-Ballesteros García R, Zamarrón Casinello MD, López Bravo MD, Molina Martínez MÁ, Díez Nicolás J, Montero López P, et al. Envejecimiento con éxito: criterios y predictores. Psicothema. 2010;22(4):641–7. 21044491

[65] Börsch-Supan A. Survey of Health, Ageing and Retirement in Europe (SHARE) Wave 6. Release version: 7.1.0. SHARE-ERIC. Data set. 2019. Available from: http://www.share-project.org/data-documentation/waves-overview/wave-6.html. doi: 10.6103/SHARE.w6.710

[66] Malter F, Börsch-Supan A, editors. SHARE Wave 6: Panel innovations and collecting Dried Blood Spots. München: Munich Center for the Economics of Aging (MEA), the Max Planck Institute for Social Law and Social Policy (MPISOC); 2017. Available from: http://www.share-project.org/uploads/tx_sharepublications/201804_SHARE-WAVE-6_MFRB.pdf

[67] Börsch-Supan A, Jürges H, editors. The Survey of Health, Aging, and Retirement in Europe–Methodology. Mannheim: Research Institute for the Economics of Aging (MEA); 2005. Available from: http://www.share-project.org/uploads/tx_sharepublications/SHARE_BOOK_METHODOLOGY_Wave1.pdf

[68] MEA- Munich Center for the Economics of Aging. The Survey of Health, Ageing and Retirement in Europe (SHARE). Release Guide 6.1.0. Mannheim: Mannheim Research Institute for the Economics of Aging (MEA); March 29 2018. 84 p. Available from: http://www.share-project.org/fileadmin/pdf_documentation/SHARELIFE_release_guide_6.1.0.pdf

[69] Malter F, Schuller K, Börsch-Supan A. SHARE Compliance Profiles–Wave 6. München: Munich Center for the Economics of Aging (MEA), the Max Planck Institute for Social Law and Social Policy (MPISOC); 2016. Available from: http://www.share-project.org/fileadmin/pdf_documentation/SHARE_Wave6_ComplianceProfiles_v8.pdf

[70] Doyal L, Gough I. A theory of human needs. Critical Social Policy. 1984;4(10):6–38. doi: 10.1177/026101838400401002

[71] Maslow AH. A theory of human motivation. Psychological Review. 1943;50(4):370–96. doi: 10.1037/h0054346

[72] Hyde M, Wiggins RD, Higgs P, Blane DB. A measure of quality of life in early old age: The theory, development and properties of a Needs Satisfaction Model (CASP-19). Aging & Mental Health. 2003;7(3):186–94. doi: 10.1080/1360786031000101157 12775399

[73] Howel D. Interpreting and evaluating the CASP-19 quality of life measure in older people. Age and Ageing. 2012;41(5):612–7. doi: 10.1093/ageing/afs023 22391614 PMC3693476

[74] Ayala A, Rodríguez-Blázquez C, Calderón-Larrañaga A, Beridze G, Teixeira L, Araújo L, et al. Influence of Active and Healthy Ageing on Quality of Life Changes: Insights from the Comparison of Three European Countries. International Journal of Environmental Research and Public Health. 2021;18(8):4152. doi: 10.3390/ijerph18084152 33919964 PMC8070976

[75] Litwin H, Stoeckel KJ. Social Network, Activity Participation, and Cognition: A Complex Relationship. Research on Aging. 2016;38(1):76–97. doi: 10.1177/0164027515581422 25878191

[76] Šimkovic M, Träuble B. Robustness of statistical methods when measure is affected by ceiling and/or floor effect. PLoS ONE. 2019;14(8):e0220889. doi: 10.1371/journal.pone.0220889 31425561 PMC6699673

[77] Hahs-Vaughn DL. A Primer for Using and Understanding Weights With National Datasets. The Journal of Experimental Education. 2005;73(3):221–48. doi: 10.3200/JEXE.73.3.221–248

[78] Hair JFJ, Anderson RE, Tatham RL, Black WC. Multivariate data analysis. Upper Saddle River, New Jersey: Prentice-Hall; 1998. 730 p.

[79] Husson F, Josse J. Multiple Correspondence Analysis. In: Blasius J, Greenacre M, editors. Visualization and Verbalization of Data. New York: Chapman and Hall/CRC; 2014. p. 163–81. doi: 10.1201/b16741

[80] Di Franco G. Multiple correspondence analysis: one only or several techniques? Quality & Quantity. 2015;50(3):1299–315. doi: 10.1007/s11135-015-0206-0

[81] Rodriguez-Sabate C, Morales I, Sanchez A, Rodriguez M. The Multiple Correspondence Analysis Method and Brain Functional Connectivity: Its Application to the Study of the Non-linear Relationships of Motor Cortex and Basal Ganglia. Front Neurosci. 2017;11(345). doi: 10.3389/fnins.2017.00345 28676738 PMC5477566

[82] Zaidi A, Gasior K, Hofmarcher MM, Lelkes O, Marin B, Rodrigues R, et al. Active Ageing Index 2012. Concept, Methodology and Final Results. Vienna: European Centre Vienna; 2013. Available from: https://www.euro.centre.org/downloads/detail/1401/1

[83] Hank K. How "successful" do older Europeans age? Findings from SHARE. The Journals of Gerontology, Series B: Psychological Sciences and Social Sciences. 2011;66B(2):230–6. doi: 10.1093/geronb/gbq089 21135069 PMC3041975

[84] Fernández-Ballesteros R, Molina M-A, Schettini R, Santacreu M. The semantic network of aging well. In: Robine J-M, Jagger C, Crimmins EM, Robine J-M, Jagger C, Crimmins EM, editors. Annual review of gerontology and geriatrics, Vol 33: Healthy longevity: A global approach. New York, NY, US: Springer Publishing Co; 2013. p. 79–107.

[85] Li CM, Lin CH, Li CI, Liu CS, Lin WY, Li TC, et al. Frailty status changes are associated with healthcare utilization and subsequent mortality in the elderly population. BMC Public Health. 2021;21(645). doi: 10.1186/s12889-021-10688-x 33794860 PMC8017879

[86] Jayanama K, Theou O, Godin J, Cahill L, Rockwood K. Association of fatty acid consumption with frailty and mortality among middle-aged and older adults. Nutrition. 2020;70:110610. doi: 10.1016/j.nut.2019.110610 31743811

[87] Stott DJ. Alcohol and mortality in older people: understanding the J-shaped curve. Age and Ageing. 2020;49(3):332–3. doi: 10.1093/ageing/afaa027 32343789

[88] Adams KB, Leibbrandt S, Moon H. A critical review of the literature on social and leisure activity and wellbeing in later life. Ageing and Society. 2011;31(4):683–712. doi: 10.1017/s0144686x10001091

[89] Arpino B, Solé-Auró A. Education Inequalities in Health Among Older European Men and Women: The Role of Active Aging. Journal of Aging and Health. 2017;31(1):185–208. doi: 10.1177/0898264317726390 28823184

[90] Boerio P, Garavaglia E, Gaia A. Active ageing in Europe: are changes in social capital associated with engagement, initiation and maintenance of activity in later life? Ageing & Society. 2021:1–19. doi: 10.1017/s0144686x21001021

[91] Lakomý M. Differences in social participation of older adults across European welfare regimes: Fourteen years of SHARE data collection. International Sociology. 2021;36(6):906–25. doi: 10.1177/0268580921993326

[92] Rodríguez-Rodríguez V. Comprensión del envejecimiento activo según contextos. In: Fernández-Mayoralas G, Rojo-Pérez F, editors. Envejecimiento Activo, Calidad de Vida y Género Las miradas académica, institucional y social. Valencia: Tirant lo Blanch; 2021. p. 127–74. Available from: https://editorial.tirant.com/co/libro/envejecimiento-activo-calidad-de-vida-y-genero-9788418329142

[93] Golinowska S, Sowa A, Deeg D, Socci M, Principi A, Rodrigues R, et al. Participation in formal learning activities of older Europeans in poor and good health. European Journal of Ageing. 2016;13(2):115–27. doi: 10.1007/s10433-016-0371-6 27358603 PMC4902828

[94] Wongsala M, Anbacken EM, Rosendahl S. Active ageing—perspectives on health, participation, and security among older adults in northeastern Thailand—a qualitative study. BMC Geriatrics. 2021;21(1):41. doi: 10.1186/s12877-020-01981-2 33430777 PMC7802255

[95] Burr JA, Mutchler JE, Caro FG. Productive activity clusters among middle-aged and older adults: intersecting forms and time commitments. The Journals of Gerontology, Series B: Psychological Sciences and Social Sciences. 2007;62(4):S267–S75. doi: 10.1093/geronb/62.4.s267 17673540

[96] Pérez Díaz J, Abellán García A, Aceituno Nieto P, Ramiro Fariñas D. Un perfil de las personas mayores en España, 2020. Indicadores estadísticos básicos. Informes Envejecimiento en Red. 2020;25:1–39. http://envejecimiento.csic.es/documentos/documentos/enred-indicadoresbasicos2020.pdf

[97] MSCBS- Ministerio de Sanidad CyBS. Encuesta Nacional de Salud de España 2017. Madrid: Ministerio de Sanidad, Consumo y Bienestar Social; 2017. Available from: https://www.mscbs.gob.es/estadEstudios/estadisticas/encuestaNacional/encuesta2017.htm

[98] Börsch-Supan A, Brugiavini A, Jürges H, Kapteyn A, Mackenbach J, Siegrist J, et al., editors. First Results from the Survey of Health, Ageing and Retirement in Europe (2004–2007). Starting the Longitudinal Dimension. Mannheim: Mannheim Research Institute for the Economics of Aging (MEA); 2008. Available from: http://www.share-project.org/uploads/tx_sharepublications/BuchSHAREganz250808.pdf

[99] Rodríguez-Rodríguez V, Rojo-Pérez F, Fernández-Mayoralas G, Ahmed-Mohamed K, Lardiés-Bosque R, Prieto-Flores ME, et al. Recursos económicos y calidad de vida en la población mayor. Revista Internacional de Sociología. 2011;69(1):195–227. doi: 10.3989/ris.2009.11.26

[100] Börsch-Supan A, Bristle J, Andersen-Ranberg K, Brugiavini A, Jusot F, Litwin H, et al., editors. Health and socio-economic status over the life course First results from SHARE Waves 6 and 7. Olderbourg: De Gruyter Publishers; 2019. doi: 10.1515/9783110617245

[101] Chansarn S. Active ageing of elderly people and its determinants: Empirical evidence from Thailand. Asia-Pacific Social Science Review. 2012;12(1):1–18.

[102] Lai CKY, Chan EA, Chin KCW. Who are the healthy active seniors? A cluster analysis. BMC Geriatrics. 2014;14(127):1–7. doi: 10.1186/1471-2318-14-127 25443864 PMC4265530

[103] Yang Y, Meng Y, Dong P. Health, Security and Participation: A Structural Relationship Modeling among the Three Pillars of Active Ageing in China. International Journal of Environmental Research and Public Health. 2020;17(19):7255. doi: 10.3390/ijerph17197255 33020396 PMC7579513

[104] Mandl B, Millonig A, Friedl V. The Variety of the Golden Agers: Identifying Profiles of Older People for Mobility Research. Transportation Research Board 92nd Annual Meeting; Washington, DC: TRB committee ANB60 Safe Mobility of Older Persons; 2013.

[105] da Silva Sousa NF, de Azevedo Barros MB. Level of active aging: Influence of environmental, social and health-related factors. Archives of Gerontology and Geriatrics. 2020;90:104094. doi: 10.1016/j.archger.2020.104094 32485497

[106] Rodríguez-Rodríguez V. Participación en actividades para envejecer activamente. In: Fernández-Mayoralas G, Rojo-Pérez F, editors. Envejecimiento Activo, Calidad de Vida y Género Las miradas académica, institucional y social. Valencia: Tirant lo Blanch; 2021. p. 231–66. Available from: https://editorial.tirant.com/co/libro/envejecimiento-activo-calidad-de-vida-y-genero-9788418329142

[107] Lee HY, Jang SN, Lee S, Cho SI, Park EO. The relationship between social participation and self-rated health by sex and age: a cross-sectional survey. International Journal of Nursing Studies 2008;45(7):1042–54. doi: 10.1016/j.ijnurstu.2007.05.007 17658532

[108] Niedzwiedz CL, Richardson EA, Tunstall H, Shortt NK, Mitchell RJ, Pearce JR. The relationship between wealth and loneliness among older people across Europe: Is social participation protective? Preventive Medicine. 2016;91:24–31. doi: 10.1016/j.ypmed.2016.07.016 27471027

[109] Michèle J, Guillaume M, Alain T, Nathalie B, Claude F, Kamel G. Social and leisure activity profiles and well-being among the older adults: a longitudinal study. Aging & Mental Health. 2019;23(1):77–83. doi: 10.1080/13607863.2017.1394442 29160718

[110] Mergenthaler A, Sackreuther I, Staudinger UM. Productive activity patterns among 60–70-year-old retirees in Germany. Ageing and Society. 2019;39(6):1122–51. doi: 10.1017/S0144686X17001404

[111] Fernández-Mayoralas G, Schettini R, Sánchez-Román M, Rojo-Pérez F, Agulló MS, Forjaz MJ. El papel del género en el buen envejecer. Una revisión sistemática desde la perspectiva científica. Prisma Social. 2018(21):149–76.

[112] Solé-Auró A, Alcañiz M. Educational attainment, gender and health inequalities among older adults in Catalonia (Spain). International Journal for Equity in Health. 2016;15(1):126. doi: 10.1186/s12939-016-0414-9 27491677 PMC4973518

[113] Foster L, Walker A. Gender and Active Ageing in Europe. European Journal of Ageing. 2013;10(1):3–10. doi: 10.1007/s10433-013-0261-0 28804278 PMC5549231

[114] Del Barrio Truchado E, Marsillas Rascado S, Sancho Castiello M. Del envejecimiento activo a la ciudadanía activa: el papel de la amigabilidad. Aula Abierta. 2018;47(1):37–44. doi: 10.17811/rifie.47.1.2018.37–44

[115] Villar F, Serrat R. Aging at a development crossroad. In: Rojo-Pérez F, Fernández-Mayoralas G, editors. Handbook of Active Ageing and Quality of Life From Concepts to Applications. Cham: Springer, International Handbooks of Quality-of-Life series; 2021. p. 121–33. doi: 10.1007/978-3-030-58031-5_7

[116] Rodríguez-Rodriguez V, Rojo-Pérez F, Fernández-Mayoralas G. Active ageing in Spain: Leisure, Community Participation and Quality of Life. In: Rodriguez de la Vega L, Toscano W, editors. Handbook of Leisure, Physical Activity, Sports, Recreation, and Quality of Life. Cham: Springer International Publishing; 2018. p. 237–57. doi: 10.1007/978-3-319-75529-8_14

[117] Dykstra PA, Fokkema T. Relationships between parents and their adult children: a West European typology of late-life families. Ageing and Society. 2011;31(4):545–69. doi: 10.1017/S0144686X10001108

[118] Petrová Kafková M. Older People as Care Givers and Their Roles In Family in the Era of Active Ageing: Case of the Czech Republic. Studia Socjologiczne. 2015;2(217):49–73.

[119] Hajek A, König H-H. Which factors contribute to loneliness among older Europeans? Findings from the Survey of Health, Ageing and Retirement in Europe: Determinants of loneliness. Archives of Gerontology and Geriatrics. 2020;89:104080. doi: 10.1016/j.archger.2020.104080 32371343

[120] Puga D, Fernández-Carro C, Fernández-Abascal H. Multimorbidity, Social Networks and Health-Related Wellbeing at the End of the Life Course. In: Rojo-Pérez F, Fernández-Mayoralas G, editors. Handbook of Active Ageing and Quality of Life From Concepts to Applications. Cham: Springer, International Handbooks of Quality-of-Life series; 2021. p. 609–28. doi: 10.1007/978-3-030-58031-5_37

[121] Beridze G, Ayala A, Ribeiro O, Fernández-Mayoralas G, Rodríguez-Blázquez C, Rodríguez-Rodríguez V, et al. Are Loneliness and Social Isolation Associated with Quality of Life in Older Adults? Insights from Northern and Southern Europe. International Journal of Environmental Research and Public Health. 2020;17(22):8637. doi: 10.3390/ijerph17228637 33233793 PMC7699832

[122] Rojo-Pérez F, Fernández-Mayoralas G, Rodríguez-Rodríguez V, Prieto-Flores ME, Rojo-Abuín JM. Residential Environment of the Elderly People in Spain. Towards a Municipal Categorization. Boletín de la Asociación de Geógrafos Españoles. 2007; (43):369–74. https://bage.age-geografia.es/ojs/index.php/bage/article/view/596

[123] García Ballesteros A, Jiménez Blasco BC. Envejecimiento y urbanización: implicaciones de dos procesos coincidentes. Investigaciones Geográficas. 2016(89):58–73. doi: 10.14350/rig.47362

[124] Leeson GW. The Growth, Ageing and Urbanisation of our World. Journal of Population Ageing. 2018;11(2):107–15. doi: 10.1007/s12062-018-9225-7

[125] Phillipson C, Grenier A. Urbanization and Ageing: Ageism, Inequality, and the Future of “Age-Friendly” Cities. University of Toronto Quarterly. 2021;90(2):225–41. doi: 10.3138/utq.90.2.11

[126] Beard JR, Petitot C. Ageing and urbanization: Can cities be designed to foster active ageing? Public Health Reviews. 2010;32(2):427–50. doi: 10.1007/BF03391610

[127] Kaas L, Kocharkov G, Preugschat E. Wealth Inequality and Homeownership in Europe. Annals of Economics and Statistics. 2019;136:27–54. doi: 10.15609/annaeconstat2009.136.0027

[128] Rojo-Pérez F, Fernández-Mayoralas G, Rodríguez-Rodríguez V, Rojo-Abuín JM. The Environments of Ageing in the Context of the Global Quality of Life among Older People Living in Family Housing. In: Mollenkopf H, Walker A, editors. Quality of Life in Old Age International and Multi-disciplinary Perspectives. Dordrecht: Springer, Social Indicators Research Series, Volume 31; 2007. p. 123–50. doi: 10.1007/978-1-4020-5682-6_8

[129] García-Valdez MT, Sánchez-González D, Román-Pérez R. Envejecimiento y estrategias de adaptación a los entornos urbanos desde la gerontología ambiental [Aging and adaptation strategies to urban environments from environmental gerontology]. Estudios Demográficos y Urbanos. 2019;34(1):101–28. doi: 10.24201/edu.v34i1.1810

[130] Marsillas S, De Donder L, Kardol T, van Regenmortel S, Dury S, Brosens D, et al. Does active ageing contribute to life satisfaction for older people? Testing a new model of active ageing. European Journal of Ageing. 2017;14(3):295–310. doi: 10.1007/s10433-017-0413-8 28936139 PMC5587458

[131] Avramov D, Maskova M. Active ageing in Europe. Strasbourg: Council of Europe; 2003. Available from: https://book.coe.int/en/population-studies-series/2687-active-ageing-in-europe-volume-1-population-series-no-41.html

[132] Bramanti D, Meda SG, Rossi G. Active Ageing among the Generations: Towards an Age-Friendly Society? In: Scabini E, Rossi G, editors. Living Longer A Resource for the family an Opportunity for Society. Champaign-Illinois: Common Ground Research Networks; 2018. p. 1–34. Available from: http://hdl.handle.net/10807/127134

[133] Lee YS, Putnam M, Morrow-Howell N, Inoue M, Greenfield JC, Chen H. Consolidated Measures of Activity among Older Adults: Results of a Three Data Set Comparison. Journal of Gerontological Social Work. 2019;62(5):502–20. doi: 10.1080/01634372.2019.1582123 30786817 PMC6703977

[134] Chen YC, Putnam M, Lee YS, Morrow-Howell N. Activity Patterns and Health Outcomes in Later Life: The Role of Nature of Engagement. The Gerontologist. 2019;59(4):698–708. doi: 10.1093/geront/gny023 29659800

[135] Boudiny K. ’Active ageing’: From empty rhetoric to effective policy tool. Ageing and Society. 2013;33(6):1077–98. doi: 10.1017/S0144686X1200030X 23913994 PMC3728916

[136] Pérez Díaz J, Abellán García A. “Active Ageing”: Its Relevance from an Historical Perspective. In: Rojo-Pérez F, Fernández-Mayoralas G, editors. Handbook of Active Ageing and Quality of Later Life From concepts to applications. Cham: Springer, series International Handbooks of Quality of Life; 2021. p. 171–84. doi: 10.1007/978-3-030-58031-5_10

[137] Fernández-Mayoralas G. Seguridad, envejecimiento activo y calidad de vida. In: Fernández-Mayoralas G, Rojo-Pérez F, editors. Envejecimiento Activo, Calidad de Vida y Género Las miradas académica, institucional y social. Valencia: Tirant lo Blanch; 2021. p. 267–306. Available from: https://editorial.tirant.com/co/libro/envejecimiento-activo-calidad-de-vida-y-genero-9788418329142

[138] Scherpenzeel A, Axt K, Bergmann M, Douhou S, Oepen A, Sand G, et al. Collecting survey data among the 50+ population during the COVID-19 outbreak: The Survey of Health, Ageing and Retirement in Europe (SHARE). Survey Research Methods. 2020;14(2):217–21. doi: 10.18148/srm/2020.v14i2.7738

[139] Angelinova Angelova M. Factors affecting the active life of people aged 50 and over in Europe before and during the pandemic. Revista Inclusiones. 2021;8:62–90.

[140] Bertoni M, Celidoni M, Bianco CD, Weber G. How did European retirees respond to the COVID-19 pandemic? Economic Letters. 2021;203:109853. doi: 10.1016/j.econlet.2021.109853 PMC9754783 36540307

[141] Novais F, Cordeiro C, Câmara Pestana P, Côrte-Real B, Reynolds Sousa T, Delerue Matos A, et al. The Impact of COVID-19 in Older People in Portugal: Results from the Survey of Health, Ageing and Retirement (SHARE). Acta Médica Portuguesa. 2021;34(11):761–6. doi: 10.20344/amp.16209 34986084

[142] Scheel-Hincke LL, Ahrenfeldt LJ, Andersen-Ranberg K. Sex differences in activity and health changes following COVID-19 in Europe—results from the SHARE COVID-19 survey. European Journal of Public Health. 2021;31(6):1281–4. doi: 10.1093/eurpub/ckab096 34406382 PMC8436396

[143] SHARE-ERIC- The Survey of Health Ageing and Retirement in Europe, The European Research Infrastructure Consortium. Results of the 1st SHARE Corona Survey; Project SHARE-COVID19 Munich: SHARE-ERIC, (Project Number 101015924, Report no. 1); 2021. 36 p. doi: 10.17617/2.3356927

[144] Spitzer S, Shaikh M, Weber D. Older Europeans’ health perception and their adaptive behavior during the COVID-19 pandemic. European Journal of Public Health. 2022;32(2):322–7. doi: 10.1093/eurpub/ckab221 34978564 PMC8755393

[145] Vergauwen J, Delaruelle K, Dykstra PA, Bracke P, Mortelmans D. The COVID-19 pandemic and changes in the level of contact between older parents and their non-coresident children: A European study. Journal of Family Research. 2022;34(1):512–37. doi: 10.20377/jfr-695

[146] Nelson G, Prilleltensky I, editors. Community Psychology: In Pursuit of Liberation and Well-Being. New York: Palgrave Macmillan; 2005.

[147] Clemson L, Mackenzie L, Ballinger C, Close JCC, Cumming RG. Environmental interventions to prevent falls in community-dwelling older people: a meta-analysis of randomized trials. Journal of Aging and Health. 2008;20(8):954–71. doi: 10.1177/0898264308324672 18815408

[148] Williams-Roberts H, Jeffery B, Johnson S, Muhajarine N. The effectiveness of healthy community approaches on positive health outcomes in Canada and the United States. Social Sciences. 2016;5(1):1–21. doi: 10.3390/socsci5010003

[149] Komp K, Johansson S. Population ageing in a lifecourse perspective: developing a conceptual framework. Ageing and Society. 2016;36(9):1937–60. doi: 10.1017/S0144686X15000756

