# Supplementary material for: Active ageing profiles among older adults in Spain: A Multivariate analysis based on SHARE study
Source: PLoS One. 2022 Aug 4;17(8):e0272549. doi: 10.1371/journal.pone.0272549 (PMC9352065; doi:10.1371/journal.pone.0272549)
Supplement: S1 Table — (PDF) [file pone.0272549.s001.pdf]

S1 Table. Selected variables according to Active Ageing pillars, and personal and contextual information

Variables and categories
**Identification variables in the survey**
Calibrated cross-sectional individual weight, Wave 6.<br>A normalized weighting variable has been calculated.
Country identifier
Language of questionnaire
Person identifier (fix across modules and waves)
**Sociodemographic characteristics, residential area, wellbeing & quality of life**
(*) Age. (Higher scores indicate more years old)<br>(min-max: 51-106; mean: 67.2; SD 11.2)
(*) Gender. (1 Male, 2 Female)
(*) Years of education. (Higher scores indicate more years of education)<br>(min-max: 0-25; mean: 8.8; SD: 5.1)
Level of education (based on the ISCED 1997)<br>Variable recoded to 5 categories: 0 Level 0 – Pre-Primary education; 1 Level 1 – Primary education or first stage of basic education; 2 Level 2 – Secondary basic education (lower or second stage); 3 Levels 3-4 – Secondary education (upper & post); 4 Levels 5-6– Tertiary education (first & second). (Higher scores indicate a higher level of education).<br>(min-max: 0-4; mean: 1.7; SD: 1.2)
(*) Current job situation.<br>Meaning of codes: 1: Retired; 2: Employed or self-employed; 3: Unemployed; 4: Permanently sick; 5: Homemaker; 97: Other)
(*) Marital status.<br>Meaning of codes: 1: Married, living with spouse; 2: Registered partnership; 3: Married, not living with spouse; 4: Never married; 5: Divorced; 6: Widowed
(*) Household size. (Higher scores indicate a higher household size)<br>(min-max: 1-10; mean: 2.4; SD: 1.2)
(*) Household type.<br>Meaning of codes: 1: Household single; 2: Household in couple and others
(*) Number of children. (High values, more number of children)<br>(min-max: 0-12; mean: 2.2; SD: 1.5)
(*) Number of grandchildren. (Higher scores indicate a greater number of granchildren)<br>(min-max: 0-20; mean: 2.1; SD: 2.8)
(*) Loneliness (short version of R-UCLA Loneliness Scale). (Higher score indicate lonely)<br>(min-max: 3-9, not lonely to very lonely; mean: 3.7; SD: 1.3)
(*) Social network satisfaction. (0 Completely dissatisfied; 10 completely satisfied)<br>(min-max: 0-10; mean: 8.9; SD: 1.2)
(*) Life satisfaction. (0 Completely dissatisfied; 10 completely satisfied)<br>(min-max: 0-10; mean: 7.5; SD: 1.8)

Variables and categories
(*) CASP-12 index for quality of life and well-being. (Higher scores indicate better quality of life)<br>(min-max: 12-48; mean: 36.1; SD: 6.5)
Area of building.<br>Meaning of codes: 1: Rural areas; 2: Large/small towns; 3: A big cities/metropolitan areas
(*) Type of building (recoded to 3 categories: 1 Farm/family house/double house; 2 Building with 3+ flats or high-rise; 3 Housing with services for elderly/nursing home)
Housing tenure regime (recoded to 2 categories: 1 Owner; 2 Others -cooperative, tenant, subtenant, rent free-)

Variables and categories
**Health pillar**
Units of alcoholic beverage consumed during the last seven days.<br>New variable formed through alcohol consumption and number of units of alcoholic beverage consumed. Thus, participants reported having no alcoholic beverage during the last 7 days are computed as '0' units of alcoholic beverage during the last seven days. (Higher scores indicate more units taken)<br>(min-max: 0-70; mean: 2.6; SD: 5.6)
(*) How often per week consume dairy products (such as a glass of milk, cheese in a sandwich, a cup of yogurt or a can of high protein supplement). (Higher scores indicate a higher frequency of consume).<br>Meaning of codes: 1: Less than once a week; 2: Once a week; 3: Twice a week; 4: 3-6 times a week; 5: Every day<br>(min-max: 1-5; mean: 4.8; SD: 0.7)
(**) Ever smoked daily for a period of at least one year. (0: No; 1: Yes)
(*) How often per week consume fruits or vegetables. (Higher scores indicate a higher frequency of consume).<br>Meaning of codes: 1: Less than once a week; 2: Once a week; 3: Twice a week; 4: 3-6 times a week; 5: Every day<br>(min-max: 1-5; mean: 4.8; SD: 0.6)
(*) How often per week consume legumes, beans or eggs. (Higher scoes indicate a higher frequency of consume).<br>Meaning of codes: 1: Less than once a week; 2: Once a week; 3: Twice a week; 4: 3-6 times a week; 5: Every day<br>(min-max:1-5; mean: 3.6; SD: 0.9)
(*) How often per week consume meat, fish or poultry. (Higher scores indicate a higher frequency of consume).<br>Meaning of codes: 1: Less than once a week; 2: Once a week; 3: Twice a week; 4: 3-6 times a week; 5: Every day<br>(min-max: 1-5; mean: 4.2; SD: 0.7)
(*) Self-perceived health - US scale. (Higher scores indicate a better self-perceived health)<br>Meaning of codes: 1: Poor; 2: Fair; 3: Good; 4: Very good; 5: Excellent<br>(min-max: 1-5; mean: 2.7; SD: 1.1)
(*) Number of limitations with activities of daily living (dressing, walking, grooming, eating, transferring bed, toileting). (Higher scores indicate more ADL limitations)<br>(min-max: 0-6; mean: 0.3; SD: 1.1)
(*) Number of limitations with instrumental activities of daily living (using a map, preparing hot meal, shopping for groceries, making telephone calls, taking medications, doing work around the house or garden, managing money such as paying bills and keeping track of expenses, leaving the house independently and accessing transportation services, doing personal laundry). (Higher scores indicate more IADL limitations).<br>(min-max: 0-9; mean: 0.7; SD: 2.0)
(*) (**) Limitation with activities. (0: Not limited; 1: Limited)
(*) Number of mobility limitations with every day activities. (Higher scores indicate more mobility limitations)<br>(min-max: 0-10; mean: 1.7; SD: 2.7)
(*) Score of verbal fluency test. (High score indicate optimal verbal fluency, that is more correct words mentioned)<br>(min-max: 0-93; mean: 16.3; SD: 7.1)
(*) Score of words list learning test - trial 1 (immediate recall). (Higher scores indicate better immediate memory)<br>(min-max: 0-10; mean: 4.5; SD: 1.9)

Variables and categories
(*) Score of words list learning test - trial 2 (delayed recall). (Higher scores indicate better delayed memory)<br>(min-max: 0-10; mean: 3.1; SD: 2.0)
(*) EURO-D- 12 items depression scale (0: Not depressed to 12: Very depressed)<br>(min-max: 0-12; mean: 2.4; SD: 2.6)
(*) Number of times in last 12 months have seen/talked to medical doctor. (Higher scores indicate a higher number of times)<br>(min-max: 0-98; mean: 5.9; SD: 2.8)
(*) (**) Stay in hospital last 12 months. (0 No, 1 Yes)
(*) Number of times in last 12 months being patient in hospital. (Higher scores indicate a higher number of times)<br>(min-max: 0-10; mean: 0.2; SD: 0.7)
(*) Number of total nights stayed in hospital in last 12 months. (Higher scores indicate a higher number of nights)<br>(min-max: 0-365; mean: 1.4; SD: 7.4)
(**) Seen a dentist/dental hygienist in the last 12 months. (0: No; 1: Yes)
(*) Eyesight reading perception. (Higher score indicate a better eyesight perceived)<br>Meaning of codes: 1: Poor; 2: Fair; 3: Good; 4: Very good; 5: Excellent<br>(min-max: 1-5; mean: 2.9; SD: 0.9)
(*) Hearing perception. (Higher scores indicate a better hearing perceived)<br>Meaning of codes: 1: Poor; 2: Fair; 3: Good; 4: Very good; 5: Excellent<br>(min-max: 1-5; mean: 2.9; SD: 1.0)
(*) Number of chronic diseases (based on the list of 18 conditions/items: A heart attack including myocardial infarction or coronary thrombosis or any other heart problem including congestive heart ; High blood pressure or hypertension; High blood cholesterol; A stroke or cerebral vascular disease ; Diabetes or high blood sugar ; Chronic lung disease such as chronic bronchitis or emphysema; Cancer or malignant tumour, including leukaemia or lymphoma, but excluding minor skin cancers; Stomach or duodenal ulcer, peptic ulcer; Parkinson disease; Cataracts; Hip fracture; Other fractures; Alzheimer's disease, dementia, organic brain syndrome, senility or any other serious memory impairment; Other affective or emotional disorders, including anxiety, nervous or psychiatric problems; Rheumatoid Arthritis; Osteoarthritis, or other rheumatism; Chronic kidney disease; Other conditions, not yet mentioned). (Higher score indicate more chronic conditions)<br>(min-max: 0-10; mean: 1.9; SD: 1.6)
(**) Doctor told you had: heart attack.<br>For the proposal of this paper, diagnoses that affected 10% or more of the interviewed population were initially selected (among the list of 18 conditions/items).<br>(0: Not selected; 1: Selected)
(**) Doctor told you had: high blood pressure or hypertension<br>(0 Not selected, 1 Selected)
(**) Doctor told you had: high blood cholesterol<br>For the proposal of this paper, diagnoses that affected 10% or more of the interviewed population were initially selected (among the list of 18 conditions/items).<br>(0: Not selected; 1: Selected)

Variables and categories
(**) Doctor told you had: diabetes or high blood sugar<br>For the proposal of this paper, diagnoses that affected 10% or more of the interviewed population were initially selected (among the list of 18 conditions/items).<br>(0: Not selected; 1: Selected)
(**) Doctor told you had: rheumatoid arthritis<br>For the proposal of this paper, diagnoses that affected 10% or more of the interviewed population were initially selected (among the list of 18 conditions/items).<br>(0: Not selected; 1: Selected)
(**) Doctor told you had: osteoarthritis/other rheumatism<br>For the proposal of this paper, diagnoses that affected 10% or more of the interviewed population were initially selected (among the list of 18 conditions/items).<br>(0: Not selected; 1: Selected)
Number of drugs/medicines taken (based on the list of 14 drugs: Drugs for: high blood cholesterol; high blood pressure; coronary or cerebrovascular diseases; other heart diseases; diabetes; joint pain or for joint inflammation; other pain (e.g. headache, back pain, etc.); sleep problems; anxiety or depression; osteoporosis; stomach burns; chronic bronchitis; suppressing inflammation (only glucocorticoids or steroids); other drugs, not yet mentioned). (Higher score indicate more drugs taken)<br>(min-max: 0-10; mean: 2.1; SD: 1.9)
Help with activities for that you have problems with.<br>This variable derived from question related with participants that reported if they have or not any difficulty with activities because of a physical, mental, emotional or memory problem (in dataset is the question or variable PH049_). Those participants that did no report any difficulty in PH049_ took code 6 in this variable.<br>Meaning of codes: 1: Don't received help in my problems with activities; 5: Yes, received help in my problems with activities; 6: Don't have problems with activities / Don't need/received help)
Number of technical aids used (based on the list of 10 items: a cane or walking stick; a zimmer frame or walker; a manual wheelchair; an electric wheelchair; a buggy or scooter; special eating utensils; a personal alarm; bars, grabs, rails to facilitate movements and to keep ones balance; raised toilet seat with/without arms; incontinence pads). (Higher score indicate more number of technical aids used)<br>(min-max: 0-7; mean: 0.5; SD: 0.7)
Pain intensity scale. (High values, more pain intensity).<br>Based on the dataset variables PH084_ (card 12) (Troubled with pain) & PH085_ (pain level)<br>If in PH084_ participants reported have no troubled with pain, then 'Pain intensity scale' = 0 'No pain troubled'<br>Meaning of codes: 0: No pain troubled; 1: Mild level of pain; 3: Moderate level of pain, 5 Severe level of pain<br>(min-max: 0-5; mean: 1.5; SD: 1.8)
Number of pains referred (based on the list of 7 pains: back; hips; knees; other joints; mouth/teeth; other parts of the body, but not joints; all over)<br>(Higher score indicate more pains referred)<br>(min-max: 0-6; mean: 0.9; SD: 1.1)
Number of frailty symptoms (based on the list of 4 frailty symptoms: falling down; fear of falling down; dizziness, faints or blackouts; fatigue).<br>(Higher score indicate more frailty symptoms)<br>(min-max: 0-4; mean: 0.6; SD: 0.9)

Variables and categories
**Lifelong learning pillar**
How often attended an educational or training course the last 12 months. (Higher score indicate a higher frequency of practice)<br>If participant reported did not attend an educational or training course the last 12 months, then the frequency is computed as "Do not practice".<br>Meaning of codes: 1: Do not practice; 2: Less often; 3: Almost every month; 4: Almost every week; 5: Almost every day)<br>(min-max: 1-5; mean: 1.1; SD: 0.6)
Self-rated reading skills. (Higher score indicate a better self-rated reading skills)<br>Meaning of codes: 1: Poor; 2: Fair; 3: Good; 4: Very good; 5: Excellent<br>(min-max: 1-5; mean: 3.0; SD: 1.1)
Self-rated writing skills. (Higher score indicate a better self-rated writing skills)<br>Meaning of codes: 1: Poor; 2: Fair; 3: Good; 4: Very good; 5: Excellent<br>(min-max: 1-5; mean: 2.9; SD: 1.1)
Computer skills. (Higher score indicate a better computer skills)<br>Meaning of codes: 0: I never used a computer; 1: Poor; 2: Fair; 3: Good; 4: Very good; 5: Excellent<br>(min-max: 0-5; mean: 1.2; SD: 1.3)

Variables and categories
**Participation pillar**
(*) Number of activities performed in last year, based in 7 items. (Higher score indicate more activities practiced)<br>(min-max: 0-7; mean: 1.1; SD: 1.2)
How often done voluntary/charity work the last 12 months. (Higher score indicate a higher frequency of practice)<br>If participant reported did not do voluntary/charity work the last 12 months, then the frequency is computed as "Do not practice".<br>(1 Do not practice; 2 Less often; 3 Almost every month; 4 Almost every week; 5 Almost every day)<br>(min-max: 1-5; mean: 1.1; SD: 0.6)
How often gone to a sport/social/other kind of club the last 12 months. (Higher score indicate a higher frequency of practice)<br>If participant reported did not attend a sport/social/other kind of club the last 12 months, then the frequency is computed as "Do not practice".<br>(1 Do not practice; 2 Less often; 3 Almost every month; 4 Almost every week; 5 Almost every day)<br>(min-max: 1-5; mean: 1.4; SD: 1.0)
How often taken part in a political/community-related organization the last 12 months. (Higher score indicate a higher frequency of practice)<br>If participant reported did not take part in a political/community-related organization the last 12 months, then the frequency is computed as "Do not practice".<br>(1 Do not practice; 2 Less often; 3 Almost every month; 4 Almost every week; 5 Almost every day)<br>(min-max: 1-5; mean: 1.1; SD: 0.4)
How often read books, magazines or newspapers the last 12 months. (Higher score indicate more frequency of practice)<br>If participant reported did not read books, magazines or newspapers the last 12 months, then the frequency is computed as "Do not practice".<br>Meaning of codes: 1: Do not practice; 2: Less often; 3: Almost every month; 4: Almost every week; 5: Almost every day)<br>(min-max: 1-5; mean: 3.0; SD: 1.9)
How often did word or number games the last 12 months. (Higher score indicate more frequency of practice)<br>If participant reported did not word or number games the last 12 months, then the frequency is computed as "Do not practice".<br>Meaning of codes: 1: Do not practice; 2: Less often; 3: Almost every month; 4: Almost every week; 5: Almost every day)<br>(min-max: 1-5; mean: 1.5; SD: 1.3)
How often played cards or games such as chess the last 12 months. (Higher score indicate more frequency of practice)<br>If participant reported did not play cards or games the last 12 months, then the frequency is computed as "Do not practice".<br>Meaning of codes: 1: Do not practice; 2: Less often; 3: Almost every month; 4: Almost every week; 5: Almost every day)<br>(min-max: 1-5; mean: 1.5; SD: 1.0)
(**) Use of internet in past 7 days<br>(0: No: 1: Yes)
Frequency of participation in activities that require a vigorous physical activity (such as sports, heavy housework, or a job that involves physical labour). (Higher score indicate more frequency of practice)<br>Meaning of codes: 1: Hardly ever, or never; 2: One to three times a month; 3: Once a week; 4: More than once a week)<br>(min-max: 1-4; mean: 2.1; SD: 1.3)

Variables and categories
Frequency of participation in activities that require a moderate level of energy such as gardening, cleaning the car, or doing a walk. (Higher score indicate more frequency of practice)<br>Meaning of codes: 1: Hardly ever, or never; 2: One to three times a month; 3: Once a week; 4: More than once a week)<br>(min-max: 1-4; mean: 3.3; SD: 1.2)
(*) Scale of social connectedness (0 Low connectedness to 4 High connectedness). (Higher score indicate a higher connectedness).<br>(min-max: 0-4; mean: 2.1; SD: 0.9)<br><br>The Wave 6 generated variables module includes a measure of social connectedness – a summary scale of the social network data that has been used previously in research (Litwin & Stoeckel, 2016). The scale incorporates the five main characteristics of the social network into one composite measure in order to capture the key facets of social network resources within a single indicator.<br>These characteristics include: (1) the number of persons cited (network size); (2) the number of cited social network members living within 25 km (proximity); (3) the number of cited persons with weekly or more contact (contact frequency); (4) the number of cited persons with very or extremely close emotional ties (support); and (5) the number of different types of relationships present within the network (diversity).<br><br>The first four of these characteristics were scored as follows: 0 = 0, 1 = 1, 2 = 2 to 3, 3 = 4 to 5, and 4 = 6 to 7 persons cited.<br>The fifth characteristic reflects the number of different relationship categories [(a) spouse, (b) other family, including children, (c) friend, and (d) other] that were present in the network (0–4). For each of these individual components of the scale, the underlying assumption is that having more social network members in each category is representative of stronger network resources. Principal component factor analysis conducted on the Wave 6 sample confirmed that the 5 items in the scale loaded on a single factor. The total raw score on the scale ranged from 0 to 20. A calibrated version of the scale (sn_scale) was employed according to the following conversion: 0 = 0, 1 = 1 to 5, 2 = 6 to 10, 3 = 11 to 15, and 4 = 16 to 20. By default, survey respondents who did not identify any social network members received a score of zero.<br>Respondents who did not answer the social networks module in Wave 6 were coded as "does not apply".
(*) (****) Number of given financial gifts 250 or more. (Higher score indicate more number of given gifts)<br>(min-max: 0-3; mean: 0.2; SD: 0.5)
(*) (****) Number of given gift, good, property 5000 or more. (Higher score indicate more number of given gifts)<br>(min-max: 0-5; mean: 0.0; SD: 0.1)
(*) (****) Given help in the household (how many). (Higher score indicate more help given)<br>(min-max: 0-4; mean: 0.1; SD: 0.3)
(*) (****) Given help to others outside the household (how many). (Higher score indicate more help given)<br>(min-max: 0-3; mean: 0.1; SD: 0.5)

Variables and categories
**Security pillar**
(*) Perception of the household able to make ends meet. (Higher score indicate more easyly to make ends meet)<br>Meaning of codes: 1: With great difficulty; 2: With some difficulty; 3: Fairly easily; 4 Easily<br>(min-max: 1-4; mean: 2.7; SD: 1.1)
(*) Household net financial assets: is an indicator calculated as the sum of hgfass – liab<br>Where:<br>hgfass is the household gross financial assets: as the sum of bank accounts, bond, stock and mutual funds; savings for long-term investments.<br>liab means the financial liabilities.<br>More information in MEA, March 29 2018.<br>(High erscore indicate a higher value of the household's net financial assets)<br>(min-max: -87,994 to 600,123; mean: 16,055.8; SD: 36,145.2)
(*) Household real assets: is an indicator based on the value of main residence and the percentage of house owned, the value of own business and the share of own business, value of cars, value of other real estate, and subtracting the mortgage on main residence.<br>More information in MEA, March 29 2018.<br>(Higher score indicate a higher value of the household's real assets)<br>(min-max: -504,413 to 5,205,000; mean: 181,156.2; SD: 233,831.8)
(*) Total household expenditure: is an indicator formed by the sum of the annual rent and home-related expenditures, the annual food at home consumption, the annual food outside home consumption, the annual home produced consumption, the out-of-pocket payment for inpatient care, the out-of-pocket payment for outpatient care, the out-of-pocket payment for drugs, the out-of-pocket payment for nursing home or home care.<br>For more information see MEA, March 29 2018.<br>(Higher score indicate a higher value of the household expenditure)<br>(min-max: 0.0-55,820.0; mean: 7,307.5; SD: 4,004.4)

Variables and categories
(*) Total household income - Version B: is an indicator formed by the sum of the earnings from employment; annual old age, early retirement pensions, survivor and war pension; annual old age, early retirement pensions, survivor and war pension; annual disability pension and benefits; annual unemployment benefits and insurance; annual payment from social assistance; sickness benefits and pensions; other regular payments from private pensions; other regular payment from private transfer; earnings from self-employment; annual income from rent or sublet; annual income from other household members; interest / dividend from bank account, bond, stock, and mutual funds.<br>For more information see MEA, March 29 2018.<br>(Higher score indicate a higher value of the total household income)<br>(min-max: 0-252,000; mean: 22,441.8; SD: 26,080,1)
(*) (****) Number of received financial gifts 250 or more. (Higher score indicate more number of received gifts)<br>(min-max: 0-3; mean: 0.0; SD: 0.2)
(*) (****) Number of received gifts, goods, properties 5000 or more. (Higher score indicate more number of received gifts)<br>(min-max: 0-5; mean: 0.0; SD: 0.2)
(*) (****) Received help from others outside household (how many). (Higher score indicate more help received)<br>(min-max: 0-3; mean: 0.2; SD: 0.5)

Notes:

(*) Variable calculated by SHARE in the generated variables modules.

(**) Variables excluded because they are dichotomous and are not allowed in the factor analysis technique performed.

In the case of chronic diseases, they have been replaced by the number of diseases variable.

Related to GALI (Global Activity Limitations), other variables (ADL, IADL and mobility limitations) remained in the study.

In the case of use of hospital services, other variables were used in the study (number of times in last 12 months being patient in hospital; number of total nights stayed in hospital in last 12 months).

(***) Variable removed from the study because it was poorly represented in the factor analysis.

(****) Discarded from the study due to the high floor effect.
