# Supplementary material for: Active ageing profiles among older adults in Spain: A Multivariate analysis based on SHARE study
Source: PLoS One. 2022 Aug 4;17(8):e0272549. doi: 10.1371/journal.pone.0272549 (PMC9352065; doi:10.1371/journal.pone.0272549)
Supplement: S2 Table — (PDF) [file pone.0272549.s002.pdf]

S2 Table. Factor Analysis by Principal Component Analysis extraction method

	Components loadings (N: 5,555)								
**Variables of Health Pillar (rotation converged in 8 iterations)**	**PC-H1- Bad health**	**PC-H2- Bad functionin g**	**PC-H3- Good cognitive functioning**	**PC-H4- High use of hospital services**	**PC-H5- Good sensory health**	**PC-H6- Do not need help / Do not use of technical aids for activities**	**PC-H7- High protein diet**	**PC-H8- High green-dairy / Low alcohol consumption**	**Communalities**
Pain intensity scale	0.847								0.736
Number of pains referred	0.818								0.710
Number of drugs/medicines taken	0.693								0.631
Number of chronic diseases	0.635				-0.312				0.588
Self-perceived health - US scale	-0.622				0.374				0.646
Number of frailty symptoms	0.581	0.357							0.525
EUROD depression scale-12 items	0.555								0.455
Number of times in last 12 months have seen or talked to medical doctor	0.470			0.439					0.441
Limitations with activities of daily living (ADL)		0.885							0.853
Limitations with instrumental activities of daily living (IADL)		0.872							0.871
Mobility limitations	0.533	0.617							0.816
Score of words list learning test - trial 2 (delayed recall)			0.866						0.795
Score of words list learning test - trial 1 (immediate recall)			0.843						0.763
Score of verbal fluency test			0.633						0.481
Num. of times in last 12 months being patient in hospital				0.847					0.741
Number of total nights stayed in hospital in last 12 months				0.835					0.711
Hearing perception					0.737				0.641
Eyesight reading perception					0.643				0.545
Help with activities for that you have problems with						0.875			0.856
Number of technical aids used	0.390	0.538				-0.582			0.829
How often consume meat, fish or poultry							0.716		0.634
How often consume legumes, beans or eggs							0.670		0.534
How often consume dairy products								0.693	0.601
How often consume fruits or vegetables								0.621	0.534
Units of alcoholic beverage the last 7 days							0.425	-0.512	0.480
								**Explained variance (%):**	**65.7**

Variables of Learning Pillar (rotation converged in 3 iterations)	Components loadings (N: 5,184)		Communalities
	PC-L9- Good writing / reading / ICTs Skills	PC-L10- High educational / training involvement	
Self-rated writing skills	0.951		0.908
Self-rated reading skills	0.947		0.897
Computer skills	0.593	0.414	0.524
How often attended an educational or training course the last 12 months		0.958	0.919
		**Explained variance (%):**	**81.2**

	Components loadings (N: 5,566)					
**Variables of Participation Pillar (rotation converged in 5 iterations)**	**PC-P11- High frequency of cognitive activities performance**	**PC-P12- High frequency of physical activities performance**	**PC-P13- High frequency of social & political activities involvement**	**PC-P14- High frequency of volunteering activities performance**	**PC-P15- High social connectedness**	**Communalities**
Number of activities performed last year	0.845		0.375			0.930
How often did word or number games (such as crossword, puzzles, Sudoku) the last 12 months	0.750					0.579
How often read books, magazines or newspapers the last 12 months	0.710					0.591
How often played cards or games such as chess the last 12 months	0.564					0.391
Frequency of participation in activities that require a moderate level of energy such as gardening, cleaning the car, or doing a walk		0.819				0.697
Frequency of participation in activities that require a vigorous physical activity (such as sports, heavy housework, or a job that involves physical labour)		0.814				0.705
How often taken part in a political/community-related organization the last 12 months			0.762			0.619
How often gone to a sport/social/other kind of club the last 12 months			0.713			0.593
How often done voluntary/charity work the last 12 months				0.954		0.928
Scale of social connectedness (low to high connectedness)					0.976	0.955
					**Explained variance (%):**	**69.9**

Variables of Security Pillar (rotation converged in 4 iterations)	Components loadings (N: 5,521)			Communalities
	**PC-S16- High self-perception of household economic status**	**PC-S17- High value of household non-financial assets & expenditure**	**PC-S18- High household economic health**	
Perception of the household able to make ends meet	0.840			0.711
Household net financial assets	0.707			0.578
Household real assets		0.920		0.848
Total household expenditure	0.328	0.450		0.356
Total household income - Version B			0.982	0.971
			**Explained variance (%):**	**69.3**

Notes:
Rotated Component Matrix. Rotation method: Varimax with Kaiser Normalization.
Factor scores ± 0.300 are shown. In grey colour, secondary factor scores.
PC-H: Principal Component of Health Pillar; PC-L: Principal Component of Learning Pillar; PC-P: Principal Component of Participation Pillar; PC-S Principal Component of Security Pillar.
Components are numbered correlatively through all principal components retained in the analysis.
