# Supplementary material for: Active ageing profiles among older adults in Spain: A Multivariate analysis based on SHARE study
Source: PLoS One. 2022 Aug 4;17(8):e0272549. doi: 10.1371/journal.pone.0272549 (PMC9352065; doi:10.1371/journal.pone.0272549)
Supplement: S1 Fig — (PDF) [file pone.0272549.s003.pdf]

S3 Fig. Active Ageing profiles model and key results

	Socio-demographics features	Household / Habitat size	Subjective perceptions
Profile I: people with moderate activity 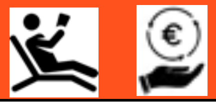	- old-old people<br>- more females<br>- widowhood<br>- low educational level	- single/small household<br>- high num. children / grandchildren<br>- rural areas/big cities	- high feeling of loneliness
Profile II: quasi-dependent persons 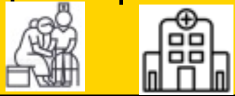	- above average age<br>- more females<br>- widowhood/never married/divorced<br>- up to lower secondary education	- relatively small household<br>- relatively num. children/grandchildren<br>- large/small towns	- high feeling of loneliness
Profile III: people with active ageing limiting conditions 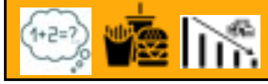	- average age<br>- more females<br>- never married/divorced<br>- low educational level	- medium household size<br>- average num. children/grandchildren<br>- large/small towns; rural areas	- relatively feeling of loneliness<br>- high level of satisfaction with social network<br>- average level of satisfaction with life<br>- average level of quality of life
Profile IV: people with diverse and balanced activity 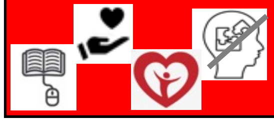	- old-young people<br>- more males<br>- married/with partner/divorced<br>- secondary education & higher	- relatively populated household<br>- having few children/grandchildren<br>- large/small towns	- no feeling of loneliness<br>- high level of satisfaction with social network<br>- high level of satisfaction with life<br>- high level of quality of life
Profile V: people with excellent active ageing conditions 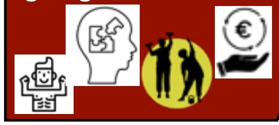	- old-young people<br>- more females<br>- married/with partner/divorced<br>- upper secondary education & higher	- small household/with couple/others<br>- low num. children/grandchildren<br>- large/small towns	- no feeling of loneliness<br>- high level of satisfaction with social network<br>- high level of satisfaction with life<br>- high level of quality of life
